# Solvent and solvation effects on reactivities and mechanisms of phospho group transfers from phosphate and phosphinate esters to nucleophiles

**DOI:** 10.3389/fchem.2023.1176746

**Published:** 2023-04-27

**Authors:** Ikenna Onyido, Onyeka F. Obumselu, Chinyelu I. Egwuatu, Nkechi H. Okoye

**Affiliations:** Department of Pure and Industrial Chemistry, Nnamdi Azikiwe University, Awka, Nigeria

**Keywords:** phospho group transfer, mechanism, solvent effects, desolvation, transition state stabilization, rate enhancements, solvent polarity

## Abstract

Organophosphorus esters fulfil many industrial, agricultural, and household roles. Nature has deployed phosphates and their related anhydrides as energy carriers and reservoirs, as constituents of genetic materials in the form of DNA and RNA, and as intermediates in key biochemical conversions. The transfer of the phosphoryl (PO_3_) group is thus a ubiquitous biological process that is involved in a variety of transformations at the cellular level such as bioenergy and signals transductions. Significant attention has been paid in the last seven decades to understanding the mechanisms of uncatalyzed (solution) chemistry of the phospho group transfer because of the notion that enzymes convert the dissociative transition state structures in the uncatalyzed reactions into associative ones in the biological processes. In this regard, it has also been proposed that the rate enhancements enacted by enzymes result from the desolvation of the ground state in the hydrophobic active site environments, although theoretical calculations seem to disagree with this position. As a result, some attention has been paid to the study of the effects of solvent change, from water to less polar solvents, in uncatalyzed phospho transfer reactions. Such changes have consequences on the stabilities of the ground and the transition states of reactions which affect reactivities and, sometimes, the mechanisms of reactions. This review seeks to collate and evaluate what is known about solvent effects in this domain, especially their effects on rates of reactions of different classes of organophosphorus esters. The outcome of this exercise shows that a systematized study of solvent effects needs to be undertaken to fully understand the physical organic chemistry of the transfer of phosphates and related molecules from aqueous to substantially hydrophobic environments, since significant knowledge gaps exist.

## 1 Introduction

### 1.1 Importance and classes of organophosphorus esters

Organophosphorus esters are widely used as pesticides, flame retardants and plasticizers in a number of household and industrial products, and as additives for lubricants and hydraulic fluids (Johnson and Henschler, 1975; Marklund et al., 2005; Wan et al., 2017). The transfer of the phosphoryl group (PO_3_) from phosphate esters to nucleophilic entities and fragments at the cellular level is a fundamental process in biology, which plays indispensable roles in the biochemistry of life, such as bioenergy transductions, signal transductions, and in genome processing (Knowles, 1980; Westheimer, 1987; Cleland and Hengge, 2006; Lassila, et al., 2011). It is the mechanistic aspects of the solution chemistry of these compounds, which are uncatalyzed reactions, that attention has been focussed on in our laboratories (Omakor, et al., 2001; Buncel et al., 2004; Onyido, et al., 2005a; Onyido, et al., 2005b; Kalu, et al., 2021; Kalu, et al., 2022), which forms the base for this review.

In this review, we are concerned with how solvents have influenced reactivities and mechanistic outcomes and their nuances in the reactions of organophosphorus esters with oxygen and nitrogen nucleophiles. The structures of the three classes of organophosphorus esters–phosphates and phosphorothioates (**1**–**3**), phosphonates and phosphonothioates (**4**), and phosphinates and phosphinothioates (**5**) are shown below. For phosphate esters, the alkylation state of the substrate determines whether it is a mono-, di-, or triester. Notice that, structurally, the compounds that end with -ate are P=O bonded (X = O), while those with -thioate ending are P=S bonded, i.e., one of the non-bridging oxygen atoms of the phosphoryl group has been replaced by sulphur (X = S).


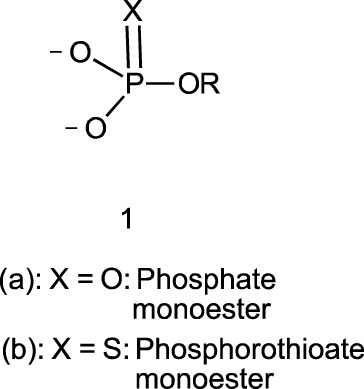



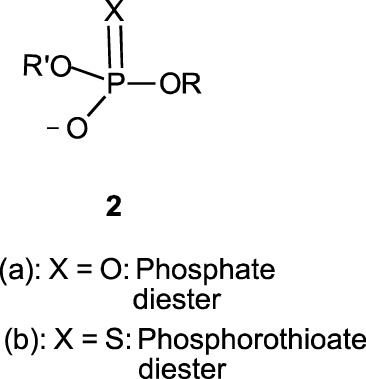



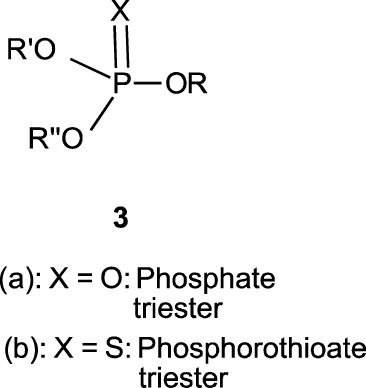



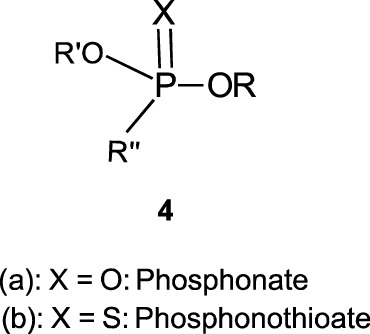



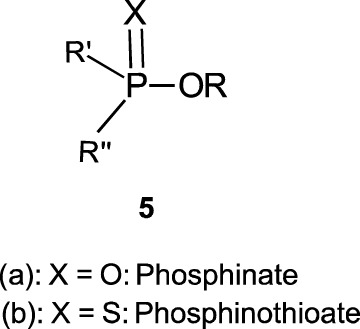


### 1.2 Phospho group transfer

Although the term “phosphoryl group transfer” refers specifically to the transfer of the phosphoryl (PO_3_) group to a nucleophilic entity in the reaction of organophosphates (**1a**-**3a**, X = O) with nucleophiles, it is loosely applied in the literature also to the transfer to nucleophiles of the other groups, PO_2_R’ (phosphonoyl group) and POR’R’’ (phosphinoyl group) from organophosphonates (**4a**, X = O) and organophosphinates (**5a**, X = O), respectively, to nucleophiles. In the same vein, the term “thiophosphoryl group transfer” for the transfer of the thiophosphoryl (PSO_2_) group from organophosphorothioates (**1b**–**3b**, X = S) to nucleophiles has also been loosely attached to the transfer of the other groups, PSOR’ and PSR’R″, from organophosphonates (**4b**, X = S) and organophosphinates (**5b**, X = S), respectively. The transferable groups from the organophosphorus esters listed as **1**–**5** above are shown in Table 1. It is clear that the distribution of charges in the transferable groups is different in all the esters shown as **1**–**5**. For this reason, we prefer to use the all-embracing term “phospho” group transfer to refer to the transfer of any of the transferable groups to nucleophiles in the span of compounds represented as **1**–**5** and reserve the term “phosphoryl” for transfers from any of the phosphates represented as **1**–**3**.

**TABLE 1:** Nomenclature of the transferable groups in organophosphorus esters 1–5

Organophosphorus ester	Name of class of esters	Transferable group to nucleophiles	Nomenclature of transferable group
1a-3a	Phosphates	PO3	Phosphoryl
1b-3b	Phosphorothioates	PSO2	Thiophosphoryl
4a	Phosphonates	PO2R’	Phosphonoyl
4b	Phosphonothioates	PSOR’	Thiophosphonoyl
5a	Phosphinates	PORR’	Phosphinoyl
5b	Phosphinothioates	PSRR’	Thiophsophinoyl

### 1.3 General and specific solvent effects

It has been long recognized that solvents can affect the course of chemical reactions conducted in them in fundamental ways (Reichardt, 1982; Buncel, et al., 2003; Reichardt and Welton, 2011). They may alter the kinetics and thermodynamics of reactions or alter product selectivities significantly by exerting substantial influences on the characteristics of the dissolved solutes (Reichardt, 2007). The origins of such interventions, and the computational tools to unravel them, have been discussed in some detail recently (Varghese and Mushrif, 2019).

As far back as 1935, Hughes and Ingold put forward their theory of solvent action (Hughes and Ingold, 1935; Ingold, 1969) as a qualitative predictive tool, based on solvent type and reactants’ charge-type, for discussing the effect of solvent polarity on the rates of bimolecular nucleophilic substitution (S_N_2) reactions. Bickelhaupt’s group (Hamlin et al., 2018) has shown that the contours of the potential energy surface (PES) of S_N_2-type processes are altered by solvation in ways that depend on the polarity of the solvent and the patterns of charge dispersal over the interacting entities. Such solvents effects, which depend on the interactions of the solutes with their environment, the solvent, and in which the solvent acts as a dielectric continuum to alter the free energies of activation ( ${∆G}^{‡}$ ) of the reaction, are collectively termed *general solvent effects* (Ingold, 1969; Parker et al., 1978; Catalán, 2009). The interactions involved in general solvent effects are generally regarded as non-specific. On the other hand, there is another group of solvent effects, termed *specific solvent (or solvation) effects*, which depend on solute-solvent interactions of the ion-dipole and acid-base types in mainly dipolar aprotic solvents (Parker, 1962; Epley and Drago, 1967; Parker, 1969) which affect the rates and mechanisms of chemical reactions executed in them. Considerable work has been achieved in determining solvent effects in nucleophilic substitution and a great variety of other reactions (Ritchie, et al., 1967; Abraham, 1986; Reichardt, 2007; Cainelli et al., 2009; Turan et al., 2022). The predictions of the effect of solvent change on such reactions have generally been borne out by published experimental results.

There exists in the literature a number of parameters by which rates of chemical reactions are correlated with various solvent properties using linear free energy relationships (Williams, 2003; Anslyn and Dougherty, 2006; Onyido, 2018). General solvent effects have been empirically parameterized (Catalán, 2009) by Kosower’s *Z* scale (Kosower, 1958a; 1958b; 1958c), Kamlet, Abboud and Taft’s dipolarity and polarizability $\pi^{*}$ scale (Taft et al., 1985), Brooker’s $\chi_{R}$ scale (Brooker et al., 1965), Dimroth-Reichardt’s $E_{T}$ (30) scale (Dimroth et al., 1963; Reichardt, 1994), Buncel’s $\pi_{azo}^{*}$ scale (Buncel and Rajagopal, 1989; 1990), *etc.* The dielectric constant of the solvent has also been deployed in correlating rates of reactions with solvent polarity (Moelwyn-Hughes, 1936a; 1936b; Laidler and Eyring, 1939; Laidler and Landskroener, 1956; Kalu, et al., 2022). On the other hand, Kamlet and Taft’s α and β scales (Kamlet and Taft, 1976a; 1976b), Arnett’s ${∆H}_{r}$ scale (Arnett, et al., 1974; 1976), Catalán’s SA and SB scales (Catalán, 2009), *etc.*, empirically parameterize specific solvent (solvation) effects.

Significant information regarding solution reactivities has become available from studies of solvent effects on diverse chemical reactions. A solvent could profoundly influence the ${∆G}^{‡}$ of a reaction in which it is conducted through its differential effects on the reactants, i.e., the ground state (GS), and the resulting transition state (TS).

### 1.4 Mechanisms of phospho transfer

**FIGURE 1:**
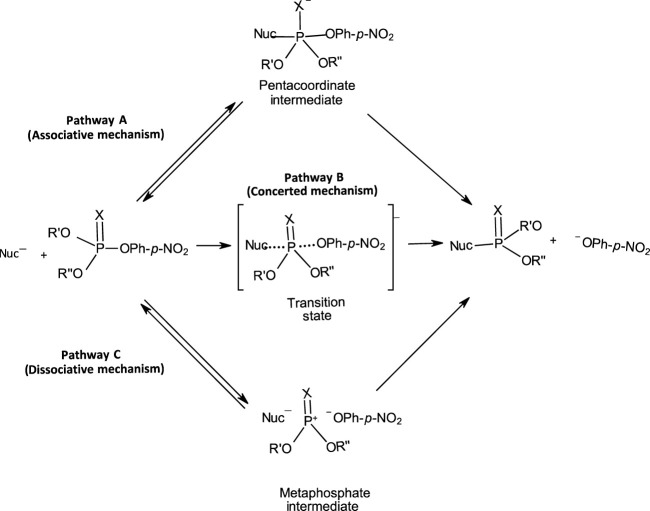
The three possible mechanisms for the reactions of nucleophiles with organophosphorus esters.

The range of mechanisms (Pathways A-C) by which organophosphorus esters of the types shown in **1**-**5** react with nucleophiles is shown in Figure 1. The substrate in this scheme is specifically a phosphate/phosphorothioate triester (**3a**/**3b**). Pathway A is the stepwise associative [A_N_ + D_N_] mechanism which involves a pentacoordinate intermediate whose formation or decomposition may be rate-limiting. Pathway B is a one-step, concerted [A_N_D_N_] S_N_2-like mechanism involving concerted bond-making and bond-rupture. Pathway C is a stepwise, dissociative [D_N_ + A_N_] mechanism in which the substrate undergoes rate-limiting ionization to yield a metaphosphate-type intermediate which reacts with the nucleophile in a fast step to yield the products.

**FIGURE 2:**
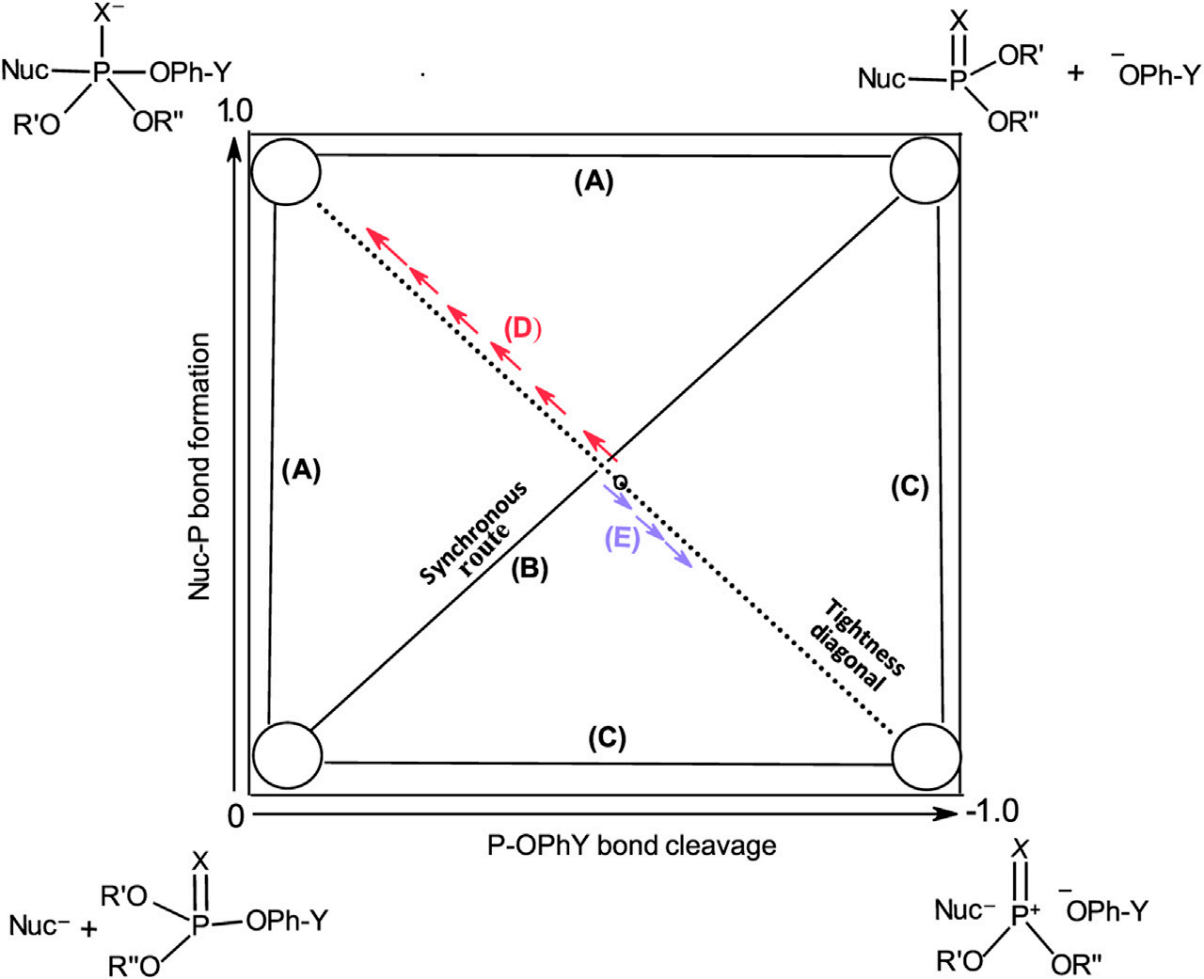
A More O’Ferrall-Jencks diagram for the reaction of a nucleophile with a hypothetical phosphate/phosphorothioate triester (**3a**/**3b**) showing the three mechanistic pathways in Figure 1 as **(A–C)**.

These three mechanisms are linked graphically in Figure 2 in a reaction map, otherwise known as the More O’Ferrall-Jencks diagram (More O’Ferrall, 1970; Jencks, 1972). In this diagram, bond fission to the leaving group is shown along the horizontal axis, while bond formation between the nucleophile and the substrate is displayed along the vertical axis. Accordingly, the stepwise mechanisms of Pathways A and C progress through the upper left and lower right corners of the diagram, respectively, while the concerted pathway (Pathway B) is defined by the diagonal linking the reactant (lower left corner) and product (upper right corner) ends of the diagram. The transition states of concerted mechanisms are located in the interior of the diagram, depending on the relative synchronicity of leaving group departure and nucleophile attack. The symmetrical concerted TS with 50% bond-making and 50% bond-breaking is located at the intersection of the synchronous route and the tightness diagonal in a wholly synchronous mechanism. The TS for the rate-limiting formation of the pentacoordinate intermediate would lie on the vertical arm of (A) while the TS for its rate-limiting decomposition would lie on the horizontal arm of (A). Route (C) is the pathway for the formation of the metaphosphate-like intermediate; its TS can only lie on the horizontal arm of (C).

### 1.5 Scope of this review

Some recent work, especially from our laboratories (Buncel et al., 2004; Onyido, et al., 2005a; Onyido, et al., 2005b; Kalu, et al., 2021; Kalu, et al., 2022), discussed in greater detail in Section 3 below, complementing earlier results in the literature (Haake et al., 1968; Douglas and Williams, 1976; Williams and Naylor, 1976; Eliseeva, et al., 1978; Istomin et al., 1982; Cook and Rahhal-Arabi, 1985; Bourne, et al., 1988; Dunn and Buncel, 1989; Dunn et al., 1990; Pregel, et al., 1995; *etc.*), demonstrates a change in the mechanism of phospho transfer from aryl phosphinates and phosphinothioates of the types shown as **6** to oxyanionic nucleophiles (HO^−^ and PhO^−^), from a concerted mechanism to an associative one, when solvent is changed from water to the less polar ethanol. Lowering the polarity of the medium by the incremental addition of ethanol to the aqueous solution (Kalu et al., 2021; Kalu et al., 2022) enabled the tracking of the loosening of the concerted TS to looser TS structures, until a solvent-independent concerted TS was attained in ethanol-rich aqueous ethanol solvents. The fact that the reactions proceed via two different mechanisms in water or aqueous ethanol solvents on the one hand and in pure ethanol solvent on the other hand, shows that the potential energy surfaces are different in the two solvent systems. In addition, the reactions became slower by factors of 10^2^–10^3^ in aqueous ethanol and pure ethanol solvents.


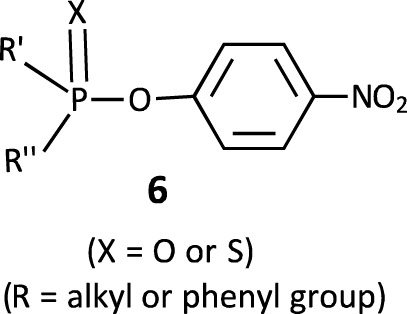


Parenthetically, it has been advocated (Herschlag and Jencks, 1987; Nikolic-Hughes et al., 2004; Leiros et al., 2004; Lassila et al., 2011) that enzymes catalyse phosphoryl group transfers from organophosphorus ester substrates by altering the dissociative structures in chemical systems to associative ones in biological processes which take advantage of induced intra-molecularity for enzymatic catalysis. This proposal is equivalent to the alteration of the PES of the reaction by the enzyme. In surveying solvent effects on mechanisms of solution phospho transfer processes as undertaken in this review, an underlying motivation was to probe the extent solvent changes influence PES changes in the reactions of organophosphorus esters with nucleophiles. It was thought that such streamlining of information could be a useful contribution to present and future discussions of the mechanisms of biological phosphoryl transfers and the related debate about the strategy through which enzymes achieve the high rate enhancements associated with biological transformations.

## 2 Solvent effects on nucleophilic reactions of phosphates and phosphinates

### 2.1 Phosphates and phosphorothioates

#### 2.1.1 Phosphate monoesters

Phosphate monoesters occur, and have recognized functions, in genetic constituents, in coenzymes, and as energy pools; they are involved in energy production and signal transduction and occur as intermediates in biochemical conversions. As products of protein phosphorylation, they play key functions in the regulation of a variety of cellular processes (Cleland and Hengge, 2006; Kamerlin et al., 2008a).

##### 2.1.1.1 Reactions with oxygen nucleophiles

**FIGURE 3:**
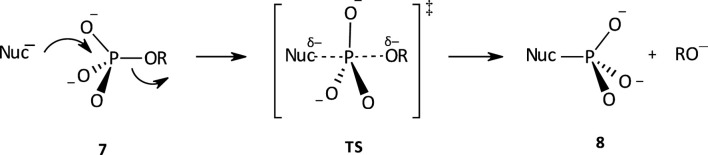
A concerted mechanism with a dissociative TS for the aqueous hydrolysis of phosphate monoester dianions.

Considerable attention has been attached to the study of the hydrolysis reactions of phosphate monoesters, across the decades (Kirby and Jencks, 1965b; Westheimer, 1987; Friedman et al., 1988; Lassila et al., 2011). Studies have shown that phosphate monoesters, reacting either as monoanions or dianions, hydrolyse via dissociative mechanisms (Benkovic and Schray, 1978; Schwartz and Murray, 2011). The phosphate monoester dianion reaction occurs via a concerted mechanism with a highly dissociative TS (see Figure 3). This TS is a loose one, in which there is extensive bond fission to the leaving group and a small amount of bond formation to the nucleophile. The characterization of the mechanism of the aqueous hydrolysis of phosphate monoester dianions as a concerted one with a highly dissociative TS is supported by small dependencies of rate on nucleophile basicity with $\beta_{nuc}$ = 0.15 (Kirby and Jencks, 1965a; Lassila et al., 2011) and large rate dependencies on leaving group basicity with $\beta_{\mathrm{lg}}$ = −1.2 (Kirby and Varvoglis, 1967), large bridge ^18^O kinetic isotope effects (Gorenstein et al., 1977; Hengge et al., 1994; Hengge and Onyido, 2005), a very small (near zero) entropy of activation (Kirby and Jencks, 1965a), and an inversion of configuration at the P centre (Buchwald et al., 1984; Friedman et al., 1988) in water. A value of $\beta_{\mathrm{lg}}$ = −0.85, measured for the hydrolysis of the alkaline phosphatase (AP)-catalyzed hydrolysis of alkyl phosphates, shows that the TS for the enzyme-catalyzed reaction is largely dissociative in character, as is the non-enzymatic reaction (O’Brien and Herschlag, 2002).

**FIGURE 4:**
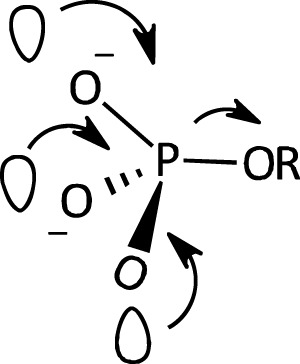
An illustration of the multiple n-σ*_P-O_ interactions which assist the rupture of the P-OR bond in phosphate monoester dianions (Medeiros et al., 2013).

A facilitating role has been ascribed (Hoff and Hengge, 1998; Medeiros et al., 2013) to the presence of the three non-bridging O atoms of the PO_3_^2-^ group in the monoester dianion; the internal electron donation interactions available in the dianion, involving the three O atoms as shown in Figure 4, provide a nucleophilic “push” which results in the elongation and consequent weakening of the P-OR bond. These interactions are thought to be responsible for the high sensitivity of the hydrolysis rates of monoester dianions to leaving group p $K_{a}$ s, as well as the higher reactivity of monoesters of phenols with p $K_{a}$ s < 5.5.

**FIGURE 5:**
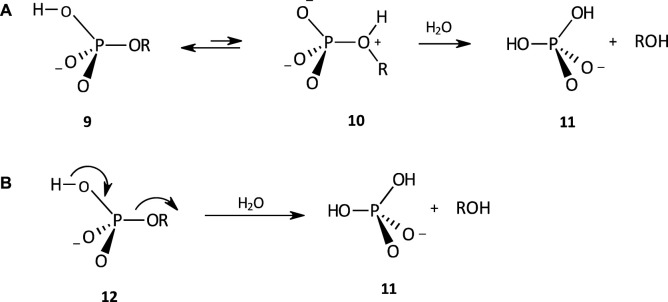
The hydrolysis of a phosphate monoester monoanion involving **(A)** an internal proton transfer pre-equilibrium to generate the dipolar species **10**, from which ROH, a powerful leaving group, is expelled, and **(B)** simultaneous proton transfer and leaving group departure, to yield **11**.

The hydrolysis of the phosphate monoester monoanion **9** proceeds by a dissociative mechanism (see Figure 5) with a negative value of ${∆S}^{‡}$ = −4.5 e.u. (Hoff and Hengge, 1998), via an initial proton transfer pre-equilibrium to yield the highly reactive dipolar species **10**. Attack of H_2_O at the P centre of **10** leads to the facile ejection of ROH as a very good leaving group. This accounts for the rate maximum observed at ∼ pH 4 in the pH-rate profiles for the hydrolysis of phosphate monoesters. Proton transfer is simultaneous with leaving group (-OR) departure as shown in **12** if the p $K_{a}$ of -OR < p $K_{a}$ of phenol (Kirby and Varvoglis, 1967), in which case proton transfer becomes partially rate-limiting. The magnitude of the $\beta_{\mathrm{lg}}$ = −0.27 measured (Kirby and Varvoglis, 1967) for monoanion hydrolysis is consistent with protonation of the leaving group in the TS. The mechanism of the hydrolysis of 2,4-dinitrophenyl phosphate monoanion has been interpreted (Admiraal and Herschlag, 2000) as a concerted one, although a mechanism with a discrete monophosphate intermediate in a preassociative mechanism cannot be ruled out (Hengge and Onyido, 2005).

Theoretical calculations by Warshel’s group reproduce linear free energy relationships (LFER) results for the solution hydrolysis of phosphate monoester dianions leaving group (Klӓhn et al., 2006). The results demonstrate that decreasing the leaving group $pK_{a}$ changes the TS character of the reaction, from associative to dissociative, and show that criteria such as LFER slopes and isotope effects do not conclusively discriminate between two TS classes. This outcome highlights the necessity for collaboration between experimental and theoretical work in discussing mechanisms and transition states of phospho transfer reactions.

In terms of relative reactivities in the hydrolysis of aryl phosphate monoester dianions and monoanions, dianions with very good leaving groups react faster than their corresponding monoanions. However, as the leaving group p*K*_*a*_ increases, i.e., the leaving group becomes poorer, the hydrolysis of the monoanions becomes faster due to the protonation of the leaving group by the proton from the phosphoryl group. Thus, there is an intersection of the reactivity of the dianion and monoanion at p*K*_*a*_ = 5.5 at 39°C (Di Sabato and Jencks, 1961; Kirby and Varvoglis, 1967; Forconi, 2015). By contrast, the monoanions of alkyl phosphates react faster in water than their dianion counterparts by a factor of ∼ 10^10^ (Lad et al., 2003). This dramatic difference in the hydrolytic reactivity of the two different ionization states of the monoesters has been ascribed to catalysis of the departure of the alkoxide leaving group by the intramolecular hydroxy group, assisted by intervening water molecules from the bulk solvent (Butcher and Westheimer, 1955; Stockbridge and Wolfenden, 2009).

Solvent effects on the rates of the solvolysis of phosphate monoester monoanion and dianion have been reported. Solvolysis of the monoanion **9** is 14 and 16 times faster in water (Kirby and Varvoglis, 1967) than in the less polar *tert*-butyl alcohol and *tert*-amyl alcohol (Hoff and Hengge, 1998). The dielectric constants, ε , of *tert*-butyl alcohol and *tert*-amyl alcohol at 25°C are 11.6 (Simonsen and Washburn, 1946), and 5.8 (Maryott and Smith, 1951), respectively. These reactions are slower in the non-polar solvents due to increased activation enthalpies in the latter solvents (Hoff and Hengge, 1998).

On the other hand, solvolysis of the dianion **7** is 7.5 × 10^3^ and 8.8 × 10^3^ times faster in *tert*-butyl alcohol and *tert*-amyl alcohol (Hoff and Hengge, 1998), respectively, than in water (Kirby and Varvoglis, 1967). Such rate enhancements may be ascribed to the reduced capacity for hydrogen bonding by the alcohol solvents when compared with water. The huge magnitudes of the solvent effects suggest that the dianion reacts by a different mechanism in the significantly less polar alcohol solvents. The activation entropy for the solvolysis of the dianion in water is 3.5 e.u. The higher values of activation entropy of 24.5 and 23.0 e.u. in *tert*-butyl alcohol and *tert*-amyl alcohol, respectively, are consistent with a change in mechanism, from A_N_D_N_ (bimolecular, S_N_2(P)-type) to D_N_ + A_N_ [a two-step, unimolecular, S_N_1(P)-type] mechanism in which there is no nucleophilic participation in the rate-determining step, with change in solvent. The change in mechanism is consistent with an altered PES, caused by a change in solvent polarity.

The case for a symmetrically solvated, diffusible monomeric metaphosphate intermediate in the reaction of the dianion of phosphate monoester was made by the study of Knowles and co-workers (Friedman et al., 1988). This was shown in the solvolysis of the dianion of chiral *p*-nitrophenyl (*R*)-[^16^O, ^17^O, ^18^O]phosphate in *tert*-butyl alcohol, which gave completely racemic *tert*-butyl [^16^O, ^17^O, ^18^O]phosphate as the product. Heavy atom kinetic isotope effect measurements ^15^ *k*, ^18^ *k*_bridge_, and ^18^ *k*_non-bridge_ indicate similar late TS structures with little or no change in bond order between P and the non-bridge O atoms for the reaction of the dianion in water and in *tert*-butyl alcohol (Hengge et al., 1994). This may seem confusing at first when it is realized that reactions in the two solvents occur by different mechanisms, A_N_D_N_ in water and D_N_ + A_N_ in *tert*-butyl alcohol. Such misconception is easily reconciled by the fact that the A_N_D_N_ mechanism in water has a highly dissociative TS with small P-nucleophile bond formation, $\beta_{nuc}$ = 0.15, and extensive P-O bond rupture, $\beta_{\mathrm{lg}}$ = −1.2 (*vide supra*). LFER studies show that the TS for the solvolysis of monoanionic phosphorylated pyridines (Herschlag and Jencks, 1989a) and the reactions of these substrates with anionic nucleophiles (Herschlag and Jencks, 1989b) is dissociative and metaphosphate-like, without the formation of a discrete intermediate. Monoanionic phosphorylated pyridines, however, are not good models for monoanionic phosphate esters because their phosphoryl group is in the dianionic form (Hengge and Onyido, 2005).

There is information on the effect of dipolar aprotic solvents as co-solvents on the rates of phosphoryl transfer from the monoanion and dianion of phosphate monoesters. For example, use of >95% aqueous dimethyl sulphoxide (DMSO) instead of water as solvent causes about 10^6^–10^7^-fold increase in the hydrolysis rate of the dianion of *p*-nitrophenyl phosphate monoester (Abell and Kirby, 1986). The dipolar aprotic solvent hexamethylphosphoramide (HMPA) produces similar accelerations. Such dramatic accelerations have been ascribed to significant desolvation of the phosphoryl group in the ground state and TS of the reaction due to the reduction in hydrogen bonding when this group binds. These GS and TS effects combine to reduce the activation free energy of the reaction significantly. Reduced solvation of the substrate is also thought to lead to a weakening of the P-O bond. The combined effect of desolvation of the ground state, stabilization of the TS, and the weakening of the P-O bond is responsible for the dramatic rate accelerations reported. In a related study, Beltran et al. (1985) showed that the hydrolysis of *p*-nitrophenyl phosphate dianion in acetonitrile (MeCN) containing 0.02 M water occurs >10^6^ times faster than the same reaction in pure water. This large rate enhancement is entropic in origin. There are other examples in which the hydrolysis of aryl phosphate dianions in water is significantly accelerated by the addition of a number of organic cosolvents which includes alcohols, acetone, dioxane, and polar aprotic solvents such as MeCN, DMSO, formamide and dimethylformamide (Kirby and Varvoglis, 1967). Although no logical relationship was found between solvent polarity parameters such as dielectric constant or the Grunwald-Winstein *Y* value and the magnitudes of the rate enhancement, the effects due to dipolar aprotic solvents were more dramatic.

The effect of added DMSO on the hydrolysis of phosphate monoesters was investigated in greater detail by Hengge and co-workers (Grzyska et al., 2002). Activation parameter and heavy atom kinetic isotope effect measurements and theoretical calculations were undertaken for the hydrolysis of a variety of phosphate monoesters. Accelerations by DMSO were restricted to phosphate monoesters dianions with p*K*_*a*_s < p*K*_*a*_ of phenol. Loss of solvation by the anionic phosphoryl group led to the weakening P-O bond; the latter was corroborated by the lower activation enthalpies and the heavy atom kinetic isotope effects (^18^ *k*_bridge_ and ^15^ *k*) measured in the study. These kinetic isotope effects show that the TS is loose in both solvent systems, supporting the fact that the observed rate accelerations in DMSO have no mechanistic change component and implications.

The response of the hydrolysis of the monoanions of phosphate monoesters to the presence of dipolar aprotic cosolvents is different from that of the dianions, as shown in the rate of the hydrolysis of the monoanion of *p*-nitrophenyl phosphate in water which becomes slower in 80% DMSO-20% aqueous formate buffer, pH = 3.2 (Abell and Kirby, 1986). In this instance, acceleration of the breakdown of the dipolar intermediate **10** by dipolar aprotic solvents is outweighed by the unfavourable proton transfer preequilibrium which is disfavoured in such solvents. It should be pointed out, however, that only the rates were affected in the examples cited above; the mechanisms of hydrolysis remained the same in pure water and in the aqueous dipolar aprotic solvents.

The hydrolysis of neopentyl phosphate monoester, an unactivated alkyl substrate when compared with the aryl phosphate monoesters discussed above, was investigated by Wolfenden in the non-polar aprotic solvent cyclohexane (Stockbridge and Wolfenden, 2009). Hydrolysis of the dianion of neopentyl phosphate monoester, present in wet cyclohexane as its tetrabutylammonium salt, proceeds with the second-order rate constant of 8.8 × 10^−10^ M^−1^s^−1^. Comparison with the rate constant for its hydrolysis in water (3.6 × 10^−22^ M^−1^s^−1^) shows that neopentyl phosphate monoester dianion reacts faster in cyclohexane than in water by a factor of 2.5 × 10^12^. The origin of this remarkable rate acceleration is entirely entropic in nature, as seen by a comparison of the activation parameters in water ( ${∆H}^{‡}$ = 47.0 kcal mol^−1^, T ${∆S}^{‡}$ = 2.7 e.u.) with those in cyclohexane ( ${∆H}^{‡}$ = 47.6 kcal mol^-1^, T ${∆S}^{‡}$ = 14.7 kcal mol^−1^).

**FIGURE 6:**
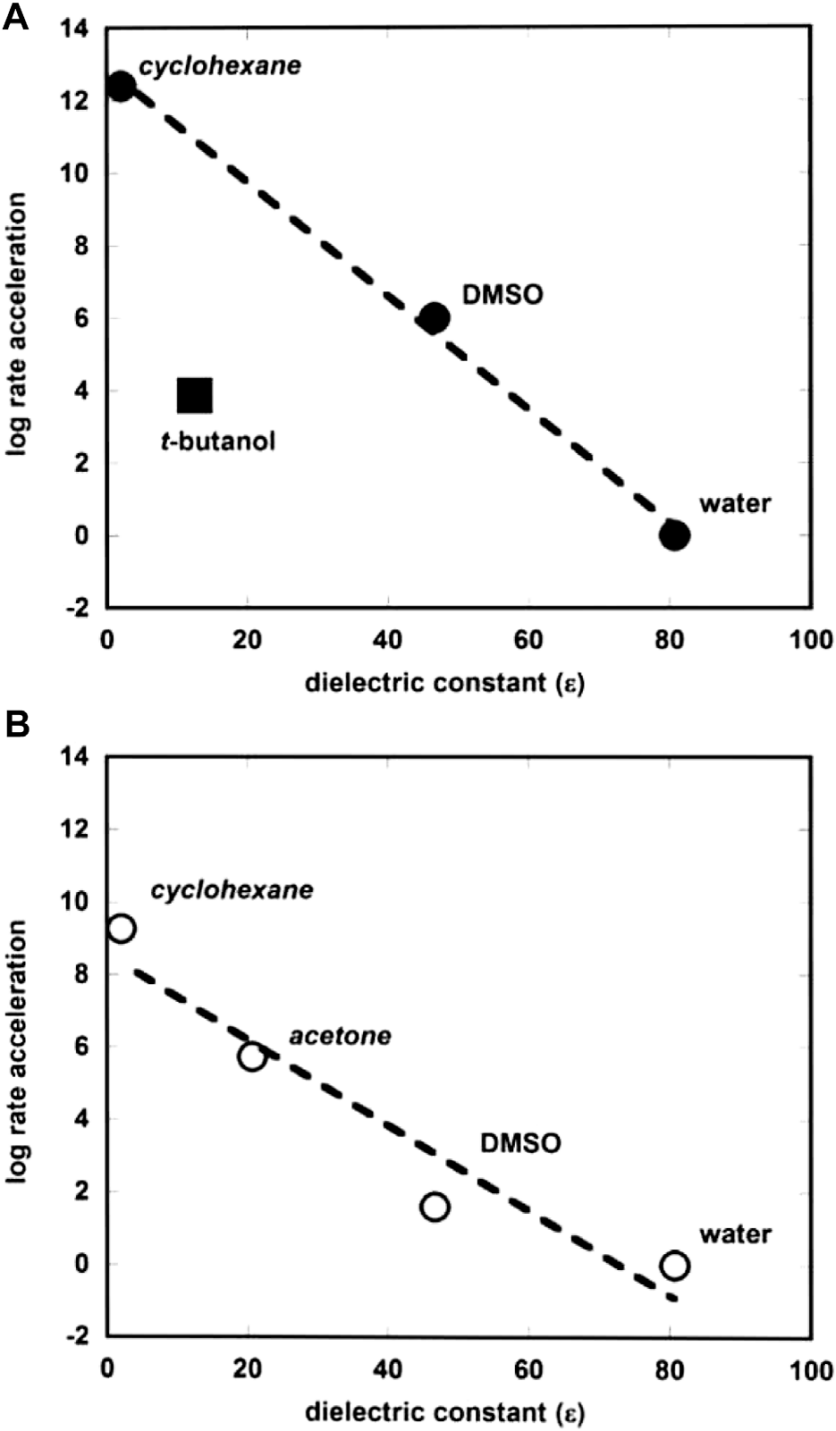
Plots of log rate acceleration *versus* dielectric constant ( ε ) of solvents for the hydrolysis of **(A)** phosphate monoester dianions and **(B)** phosphate diesters. Solvents in italics denote reactions involving alkyl phosphate substrates. Reprinted with permission of M. Forconi (2015) Adv. Phys. Org. Chem. 49, 57–101. Copyright Elsevier Ltd.

Forconi (2015) has correlated rate accelerations of the hydrolysis of alkyl and aryl phosphate monoester dianions in protic and aprotic solvents by plotting the log of rate acceleration versus solvent dielectric constant as shown in Figure 6. The plot is interestingly linear, despite the structural differences in the substrate-types. The point for *tert*-butyl alcohol shows significant deviation from the straight line, and this is thought to arise from the fact that this solvent, though non-polar, is able to form hydrogen bonds with the reactive species, which is not possible with the other solvents apart from water. The correlation seems to provide evidence for catalysis by the more hydrophobic solvents due to the differences between the electrostatic properties of the ground and transition states of the reactions. A comment is made below (see Section 2.2.1) on the similar plot (Figure 2B) for phosphate diesters.

##### 2.1.1.2 Reactions with nitrogen nucleophiles

Reactions of phosphate monoesters with amine nucleophiles have been reported. Kirby and Jencks (1965a) studied the reactions of a variety amine nucleophiles with *p*-nitrophenyl phosphate dianion in aqueous solutions. A wide range of reactivity behaviour toward this substrate was found in the amines investigated, ranging from very high reactivity, including the manifestation of an α-effect, through moderate reactivity, to no reactivity at all. An important mechanistic information obtained from this study is the very small dependence of the rate on the basicity of the attacking amine, $\beta_{nuc}$ = 0.13, indicating a displacement reaction with a small amount of bond formation in the TS. Bronsted-type plots for the reactions of pyridine, triethylenediamine, hydrazine and the protonated form of triethylenediamine with monoester dianions yielded $\beta_{\mathrm{lg}}$ values of −1.03, −0.94, −0.97, and −1.01, respectively (Grzyska et al., 2002). The overall mechanistic picture for the reaction of amines with phosphate monoester dianions in aqueous solutions conveys a concerted displacement reaction in which there is a small amount of bond formation and extensive bond rupture in the TS. Even for the reactions of unhindered tertiary amines with 2,4-dinitrophenyl phosphate dianion in which rates are independent of the basicity of the amines, i.e., $\beta_{nuc}$ ∼ 0, the second-order kinetics observed and the substantially negative ${∆S}^{‡}$ (−19 e.u.) measured show that the reactions are bimolecular (Kirby and Varvoglis, 1968). Values of $\beta_{\mathrm{lg}}$ in these reactions are in the range of −1.0 to −1.2 for the reactions with amines, as they are in hydrolysis reactions (Kirby and Jencks, 1965b; Kirby and Varvoglis, 1967; Kirby and Varvoglis, 1968; Jencks, 1980).

The situation is different with phosphate monoester monoanions. Phosphorylation of amine nucleophiles, such as pyridine, by simple phosphate monoester monoanions does not occur, whereas fluoride ion reacts with the monoanion of acetyl phosphate to give fluorophosphate; the cleavage of the P-O bond in the latter compound is catalysed by tertiary amines (Di Sabato, and Jencks, 1961). The reactions of nicotinamide with a series of monoester monoanions give a Bronsted-type plot with $\beta_{\mathrm{lg}}$ = −1.03. The reactions of substituted pyridines with 2,4-dinitrophenyl phosphate monoanion give a $\beta_{nuc}$ value of 0.56 (Grzyska et al., 2002), nearly equal to $\beta_{nuc}$ = 0.54 measured for the reactions of same nucleophiles with di-2,4-dinitrophenyl phosphate, an indication that the two reactions are similar and may proceed by a similar mechanism. The similarity of the thermodynamic parameters for the two reactions, ${∆H}^{‡}$ = 16.8 kcal mol^-1^; ${∆S}^{‡}$ = −19.4 e.u. for 2,4-dinitrophenyl phosphate monoanion versus ${∆H}^{‡}$ = 17.6 kcal mol^-1^; ${∆S}^{‡}$ = −23.1 e.u. for di-2,4-dinitrophenyl phosphate, also argues for similar mechanisms for the reactions of the monoanions and dianions. A concerted mechanism for the monoanion is satisfied by the foregoing facts.

On the other hand, Ramirez and Marecek (1979); Ramirez and Marecek (1980) have shown that the reactions of 2,4-ditrophenyl phosphate monoanion with water and alcohols in MeCN solutions undergo phosphoryl transfer by the addition-elimination, associative [A_N_ + D_N_] mechanism (Pathway A in Figure 1) involving a pentacoordinate intermediate and may be uncatalyzed or nucleophilic amine-catalysed. The reactions are slow. Steric hindrance in ROH decreases the rate of formation of alkyl phosphates significantly. There is no formation of *tert*-butyl phosphate when the reaction is performed in tert-butyl alcohol. Reactions of the dianion, on the other hand, occur by the elimination-addition, dissociative [A_N_D_N_] mechanism (Pathway C in Figure 1) involving a metaphosphate intermediate and are not subject to nucleophilic catalysis by added amine. These reactions are relatively fast and the rates of formation of alkyl phosphates are insensitive to structural variations in ROH. Reaction in the presence of *tert*-butyl alcohol produces *tert*-butyl phosphate. Thus, changing the solvent from water to MeCN changes the mechanistic profiles of the reactions of the monoanion and dianion of 2,4-dinitrophenyl phosphate.

#### 2.1.2 Phosphorothioate monoesters

The replacement of the non-bridging oxygen in phosphate esters with sulphur to obtain phosphorothioate esters changes rates of reactions. This change in rate following the change of the non-bridging O to S is termed the *thio effect*, expressed as the kinetic ratio *k*_*o*_/*k*_*s*_, where *k*_*o*_ and k_s_ are the rate constants for the reactions of the oxygen and thio substrates, respectively. Several explanations have been advanced for the difference in the reactivity of phosphorothioate and phosphate esters. These include the different electron-releasing abilities of O and S (Chlebowski and Coleman, 1974; Zhang et al., 1999), changes in polarization of P=O and P=S bonds (Ketelaar et al., 1952), the relative electrophilicity of the P atom in phosphoryl *versus* thiophosphoryl groups (Teichmann and Hilgetag, 1967; Neimysheva, et al., 1968), and structural differences between the two compounds (Cheng et al., 2002). Carvalho et al. (2015) have emphasized the interplay between nucleophile charge and TS solvation in defining reactivities, including thio effects, in the S_N_2(P) reactions of simple phosphoryl transfer systems.

The solvolyses of chiral *O*-aryl phosphorothioate monoester dianions in water and ethanol proceed by the S_N_1-type (D_N_ + A_N_) mechanism involving rate-limiting formation of free thiometaphosphate as an intermediate (Cullis and Iagrossi, 1986; Domanico et al., 1986; Cullis et al., 1987; Burgess et al., 1988). This conclusion was supported by the large degree of racemization observed in the reactions. The value of $\beta_{\mathrm{lg}}$ = −1.1 measured for these reactions (Hollfelder and Herschlag, 1995) is, however, similar to $\beta_{\mathrm{lg}}$ = −1.2 determined for their phosphate counterparts which are known to react via a concerted mechanism with highly dissociative TS. Leaving group KIEs show that leaving group rupture occurs to the same extents in the TS of both *p*-nitrophenyl phosphorothioate and its phosphate counterpart, even though these analogues react by different mechanisms (Catrina and Hengge, 1999); while the reaction of the phosphate ester involves nucleophilic participation in the TS, consistent with a concerted mechanism, the reaction of phosphorothioate ester dianion does not. Why then is the metaphosphate anion not formed in the reaction of phosphate monoester dianions but formed in the reaction of their phosphorothioate counterparts? Experimental evidence shows that the metaphosphate anion is less stable than its thiometaphosphate counterpart (Meyerson et al., 1984; Henchman et al., 1985; Roesky et al., 1986) and is formed only in the absence of a proficient acceptor. A value of $\beta_{\mathrm{lg}}$ = −0.8 was measured for the alkaline phosphatase (AP)-catalyzed hydrolysis of a series of *O*-aryl phosphorothioates, showing that the TS of the enzymatic reaction, like its non-enzymatic counterpart with $\beta_{\mathrm{lg}}$ = −1.1, has considerable dissociative character (Hollfelder and Herschlag, 1995).

Activation parameters for the hydrolysis of *p*-nitrophenyl phosphorothioate dianion were measured as ∆H^‡^ = 37.0 kcal mol^−1^, ∆S^‡^ = +29 e.u., which amount to ∆G^‡^ = 27.9 kcal mol^−1^ at 39°C (Catrina and Hengge, 1999); these may be compared with the values of ${∆H}^{‡}$ = 30.6 kcal mol^-1^, ${∆S}^{‡}$ = +3.5 e.u., and ${∆G}^{‡}$ = 29.5 kcal mol^−1^ at 39°C for the phosphate monoester dianion hydrolysis (Kirby and Jencks, 1965a). The phosphorothioate substrate reacts 12.6 times faster than its phosphate counterpart, giving rise to an inverse thio effect, *k*_*o*_/*k*_*s*_ = 0.08. The higher reactivity of the phosphorothioate substrate over its phosphate counterpart in water was ascribed to TS effects, since the solvation free energies of the two compounds are within 0.1 kcal mol^−1^, the poorer hydrogen bonding ability of S relative to O notwithstanding (Catrina and Hengge, 1999).

*p*-Nitrophenyl phosphorothioate monoester monoanion hydrolyses by the same mechanism as the phosphorothioate monoester dianion, involving the formation of a free thiometaphosphate intermediate. Activation parameters are ${∆H}^{‡}$ = 22.0 kcal mol^−1^, ${∆S}^{‡}$ = −1 e.u., and ${∆G}^{‡}$ (at 39°C) = 22.2 kcal mol^−1^ for the hydrolysis of *p*-nitrophenyl phosphorothioate (Catrina and Hengge, 1999) and ${∆H}^{‡}$ = 25.4 kcal mol^−1^, ${∆S}^{‡}$ = −4.5 e.u., and ${∆G}^{‡}$ (at 39°C) = 29.5 kcal mol^−1^ for the reaction of the corresponding phosphate monoester (Kirby and Varvoglis, 1967). The phosphorothioate substrate hydrolyses 1,380 times faster than its thio analogue, to give *k*_*o*_/*k*_*s*_ = 7.2 × 10^−4^. This remarkable difference in the reactivity of the two phosphate ester analogues is principally located in the 3.4 kcal mol^−1^ difference in ${∆H}^{‡}$ for the hydrolysis of the two substrates and a slightly favourable entropy of activation, ${∆∆S}^{‡}$ = +3.5 e.u. It has been suggested that the implication of this entropic advantage for the phosphorothioate substrate is that its reaction progresses largely through a thiometaphosphate intermediate mechanism (Catrina and Hengge, 1999), as hinted by the stereochemical findings of Harnett and Lowe (1987).

2,4-Dinitrophenyl phosphorothioate dianion undergoes hydrolysis in aqueous solution with an activation volume of 11 cm^3^ mol^−1^ (Burgess et al., 1988), as against the value of −4.8 cm^3^ mol^−1^ measured for the hydrolysis of 2,4-dinitrophenyl phosphate (Ramirez et al., 1986). These results are consistent with an S_N_1 mechanism with the formation of a free thiometaphosphate anion intermediate for the phosphorothioate substrate and a concerted mechanism for its oxygen analogue.

Solvent effects on the hydrolysis of the monoanion and dianion of *p*-nitrophenyl phosphorothioate were elucidated in aqueous DMSO solutions (Catrina and Hengge, 1999). These workers found that the log of the rate constant (s^-1^) for the hydrolysis of *p*-nitrophenyl phosphorothioate dianion was curvilinearly dependent on % DMSO in a concave upward fashion. The reaction was accelerated by more than 6 orders of magnitude in 95% DMSO compared with the reaction in water. Measured activation parameters in this solvent system of ${∆H}^{‡}$ = 22.9 kcal mol^−1^ and ${∆S}^{‡}$ = +12 e.u. may be compared with ${∆H}^{‡}$ = 37.0 kcal mol^−1^ and ${∆S}^{‡}$ = +29 e.u. obtained in water. These results show that the dramatic acceleration in 95% DMSO is enthalpic in origin, with ${∆∆H}^{‡}$ = 14.1 kcal mol^−1^, favouring reaction in aqueous DMSO and reflecting a significant stabilization of the TS by the dipolar aprotic solvent. The solvent effect on the reaction of the monoanion is much less dramatic than its effect on the dianion: the rate of the reaction of this species is in fact decreased only slightly with increasing DMSO quantities. The activation parameters for reaction of the monoanion in 95% DMSO ( ${∆H}^{‡}$ = 18.8 kcal mol^−1^ and ${∆S}^{‡}$ = −12 e.u.) when compared with values in water ( ${∆H}^{‡}$ = 22.0 kcal mol^−1^ and ${∆S}^{‡}$ = −1 e.u.) show that the reduction of ${∆H}^{‡}$ by 3.2 kcal mol^-1^ in the aqueous DMSO solvent is not sufficient to counterbalance the less favourable ${∆S}^{‡}$ (by 11 e.u.) in this solvent. The net effect is that ${∆G}^{‡}$ in 95% DMSO is 0.5 kcal higher than the value in water, leading to slight decrease in rate in the former solvent.

Sorensen-Stowell and Hengge (2006) investigated the thermodynamic origin for the accelerated hydrolysis rate of the dianions of phosphate and phosphorothioate esters in aqueous DMSO solvent. In 0.6 (mol) aqueous DMSO (60 mol% water in DMSO), ${∆H}^{‡}$ for the hydrolysis of *p*-nitrophenyl phosphate was measured as 26.2 kcal mol^−1^, compared to the value of 30.6 kcal mol^−1^ obtained in water, while for the *p*-nitrophenyl phosphorothioate substrate, there was a reduction of ${∆H}^{‡}$ from 37.0 kcal mol^−1^ in water to 29.0 kcal mol^−1^ in the aqueous DMSO solvent. These authors explained that the two negative charges on each reactant (*p*-nitrophenyl phosphate and *p*-nitrophenyl phosphorothioate) are more dispersed in the TS which is better stabilized by the aqueous DMSO solvent than pure water, leading to significant reductions in ${∆H}^{‡}$ and accelerated rates of hydrolysis. The experimental data revealed that the origin of the reduced ${∆H}^{‡}$ in the reactions of both substrates is the more exothermic transfer of their transition states from water to the aqueous DMSO solvent than for the transfers of the respective reactants. The authors opined that additional effects could be at play at higher DMSO fractions where more dramatic rate accelerations had been reported (Abell and Kirby, 1986; Catrina and Hengge, 1999).

#### 2.1.3 Phosphate diesters

Phosphate diesters occur as nature’s genetic material in the form of DNA and RNA (Westheimer, 1987) and in phosphatidyl choline, one of the major constituents of the cell membrane (Sung, 1994). The resistance of the phosphodiester bond in DNA to hydrolysis in the absence of enzymes is well recognized. The neutral forms of this ester, **13**, whose p*K*_*a*_s are typically in the range of 1-2, exist as the monoanion **2** in the entire pH range. The negative charge of the monoanion is shared between two equivalent oxygens, thus making the P centre in **2** less electrophilic than in phosphate triesters. The absence of a proton, as found in the monoanion of phosphate monoesters, ensures that there are no other ionic forms of **2** around neutral pH (Kirby et al., 2015). The presence of enzymes, structural endowments in uncatalyzed reactions such as coordination of the phosphate diester substrate to metal ion complexes (Humphry et al., 2004; Berreau, 2006; Tirel et al., 2014), linkage of the two RO groups via 5-membered rings which induces the stereoelectronic effect (Taira et al., 1984) or ring strain (Westheimer, 1968; Kluger and Taylor, 1990), advantageously located nucleophiles (Livieri et al., 2007; Mancin and Tecilla, 2007), and inherently very good leaving groups (Kirby and Younas, 1970a) all enhance the reactivity of phosphate diester monoanions. Generally speaking, in the absence of intervening structural factors, the reactions of phosphate diester substrates occur mostly by the concerted S_N_2(P)-type mechanism (Hengge and Onyido, 2005).


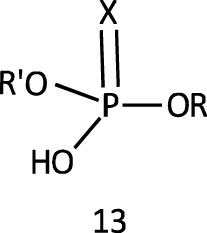


Zalatan and Herschlag (2006) have shown that both the solution (non-enzymatic) and the AP-catalyzed (enzymatic) hydrolysis of a series of methyl phenyl phosphate diesters proceed via identical concerted transition states, i.e., the TS for the enzyme-catalyzed hydrolysis is indistinguishable from that of its solution counterpart. Rather than alter the TS of the solution reaction, as advocated to explain the high rate enhancements delivered by enzymes (see Section 1.3 above), the enzyme enacts catalysis by stabilizing the solution TS.

##### 2.1.3.1 Reactions with oxygen nucleophiles

The hydrolysis of bis(*p*-nitrophenyl) phosphate, a substrate with a weakly basic leaving group, in neutral water at 50°C is too slow for direct measurement. However, measuring the rates at 80°C, 90°C and 100°C, gave the first-order rate constants as 1.0 × 10^−8^ s^−1^, 2.5 × 10^−8^ s^−1^, and 6.5 × 10^−8^ s^−1^, respectively (Chin et al., 1989). From these rate constants, the activation parameters for the reaction were obtained as ${∆H}^{‡}$ = 24.8 kcal mol^−1^, ${∆S}^{‡}$ = −25.4 e.u. Using these activation parameters, the rate constant at 50°C was calculated as 3 × 10^−10^ s^−1^. With the concentration of water in pure water = 55.5 M, the second-order rate constant for the hydrolysis of bis(*p*-nitrophenyl) phosphate in neutral water at 50°C becomes 5.4 × 10^−12^ M^−1^ s^−1^. This value may be compared with the value of 5.4 × 10^−9^ M^−1^ s^−1^ measured for the hydrolysis of 2,4-dinitrophenyl phosphate at 39°C. Although Chin et al. (1989) did not discuss the mechanism of reaction, the significantly high negative value of ${∆S}^{‡}$ points to a concerted S_N_2(P)-type mechanism. The value of Bronsted $\beta_{nuc}$ = 0.31 for the reactions of oxyanions with aryl methyl phosphate diesters (Kirby, and Younas, 1970b), a $\beta_{\mathrm{lg}}$ value of −0.97 for the hydrolysis of diaryl phosphate anions (Kirby, and Younas, 1970a), and heavy atom kinetic isotope effects measured for the alkaline hydrolysis of 3,3-dimethyl *p*-nitrophenyl phosphate (Hengge and Cleland, 1991), all point to concerted mechanisms for these hydrolysis reactions.

The alkaline hydrolysis of bis(*p*-nitrophenyl) phosphate was studied in aqueous DMSO, dioxane- and MeCN-water mixtures (Gomez-Tagle et al., 2006). In all the solvent mixtures investigated, the reaction rate showed a steady decline in the 0–70 vol% region of organic cosolvent, to reach about 50% of its value in water; thereafter the reaction rate increased sharply as the organic solvent content increased. The response of the rate constant to increasing per cent of the organic cosolvent was found to be qualitatively similar for the three organic solvents. The solvent effect in this system was satisfactorily correlated using a simplified stepwise solvent-exchange model, which focuses on the equilibrium replacement of solvent molecules in a solute’s coordination sphere in the treatment of the free energies of transfer of solutes to a mixed solvent (Waghorne, 1993; Gomez-Tagle et al., 2006). In 94% DMSO the rate constant for the hydrolysis of bis(*p*-nitrophenyl) phosphate was accelerated ∼ 15-fold relative to its value in water at 37°C; the effects of the addition of dioxane and MeCN were more modest than that of DMSO, in that order. These modest effects apparently result from a marginal solvation advantage conferred on the TS, over the GS, by the presence of these organic solvents.

Solvent effects on the hydrolysis of the unactivated phosphate diester dineopentyl phosphate in water were studied (Stockbridge and Wolfenden, 2010) by determining the rates of hydrolysis in the non-polar aprotic solvents acetone and cyclohexane and comparing them with the rate in water (Schroeder et al., 2006). The reactions in water and these solvents proceed wholly via P-O bond cleavage. The reactions proceed much more rapidly in cyclohexane and acetone than in water: at 25°C, the second-order rate constant for the hydrolysis of the substrate is increased by factors of 2 × 10^9^ and 5 × 10^5^ in cyclohexane and acetone, respectively. These results clearly show that the rate acceleration is dependent on solvent polarity, since the dielectric constants, ε , for these solvents are 2.0 at 20°C and 20.7 at 25°C for cyclohexane and acetone (Maryott and Smith, 1951), respectively. Arrhenius parameters obtained from plots constructed with second-order plots gave the values of ${∆G}^{‡}$ = 40.5 kcal mol^−1^, ${∆H}^{‡}$ = 29.5 kcal mol^−1^, and ${T∆S}^{‡}$ = −11.0 kcal mol^−1^ for water, ${∆G}^{‡}$ = 32.6 kcal mol^−1^, ${∆H}^{‡}$ = 22.6 kcal mol^−1^, and ${T∆S}^{‡}$ = −10.0 kcal mol^−1^ for acetone, and ${∆G}^{‡}$ = 27.8 kcal mol^−1^, ${∆H}^{‡}$ = 19.6 kcal mol^−1^, and ${T∆S}^{‡}$ = −8.2 kcal mol^−1^ for cyclohexane (Stockbridge and Wolfenden, 2010). The implication of these results is that the rate accelerations in the non-polar solvents are mainly enthalpic ( ${∆H}^{‡}$ ) in origin, with a minor contribution from ${T∆S}^{‡}$ , associated with solvation changes in the GS and the TS. Unactivated phosphate diesters undergo hydrolysis in water via the associative mechanism (Kamerlin et al., 2008b); the supposition that the reactions in cyclohexane and acetone occurs by the same mechanism is justified by the significantly high negative ${∆S}^{‡}$ values of −33.6 e.u. in cyclohexane (Stockbridge and Wolfenden, 2010) and −27.5 e.u. in water. Just as done for alkyl and aryl phosphate monoester dianions, Forconi (2015) correlated rates of the hydrolysis of alkyl and aryl phosphate diesters with the dielectric constants of the solvents (Figure 6B), which suggests a possible role for desolvation in the rate increases obtained in solvents of lower polarity than water.

#### 2.1.4 Phosphate triesters

Although phosphate esters are not naturally occurring, their worldwide deployment as pesticides (Wan et al., 2017) and their existence as stockpiles of nerve agents (Kim et al., 2011) make the study of the strategies and mechanisms for their detoxification and degradation an important chemical enterprise. For the reason that phosphate triesters are constitutionally neutral and do not repel nucleophiles as their monoester and diester counterparts do, these substrates are the most reactive among the three classes of phosphate esters (Hengge and Onyido, 2005). Although LFER studies show that the reactivity of phosphate triesters depends on the nature of the nucleophile and the leaving group, the nature of the two non-leaving groups, called spectator groups, can also be an important factor (Kirby et al., 2011; Mora et al., 2012; Kirby et al., 2013). There is evidence that triesters react by mainly associative mechanisms involving the phosphorane intermediate (Cleland and Hengge, 1995; Kirby and Nome, 2015), although concerted mechanisms have been reported with less nucleophilic bases such as phenoxides, secondary alicyclic amines and pyridines (Ba-Saif et al., 1990; Omakor et al., 2001; Castro et al., 2011). A theoretical study of the alkaline hydrolysis of dimethyl phosphate triesters showed that substrates with poor leaving groups ( ${pK}_{a}$ > 8) react by the stepwise mechanism whereas esters with good leaving groups hydrolyse via the concerted mechanism (Tarrat, 2010). With the substrate dineopentyl phosphate, theoretical calculations by Kamerlin et al. (2008b) demonstrate that water attack proceeds via an associative mechanism with proton transfer to produce a phosphorane intermediate.

##### 2.1.4.1 Reactions with oxygen and nitrogen nucleophiles

Under nucleophilic catalysis by imidazole, diethyl 2,4-dinitrophenyl phosphate undergoes hydrolysis in water, first to give the phosphorylimidazole intermediate **14** in a concerted reaction, which then decomposes to products (Orth et al., 2011). The corresponding intermediate with 1-methylimidazole was unstable but its presence was inferred by NMR and mass spectral analysis (Campos et al., 2015). For the reactions of both imidazoles in DMSO-water mixtures, four distinct regions are discernible in the log rate *versus* mol% DMSO plot. The rate first decreased in the 0–10 mol% DMSO range, remained constant in the 10–20 mol% DMSO range, increased rapidly in the 20–40 mol% DMSO range and then less rapidly in the 40–100 mol% DMSO range. The rates were 10^3^-fold and 2 × 10^2^-fold higher in 100% DMSO for imidazole and 1-methylimidazole, respectively, relative to rate in pure water. The solvent effect recorded in this study (Orth et al., 2011) was satisfactorily correlated with Catalan’s SA, SB and SPP solvent parameters (Catalán et al., 2001; Gomez-Tagle et al., 2006; Catalán, 2009) which showed that decreased solute-solvent interactions in the wholly organic solvent or predominantly organic solvent in the aqueous organic solvent, caused by decreased hydrogen bonding, led to higher rates in DMSO-rich and pure DMSO solvents.


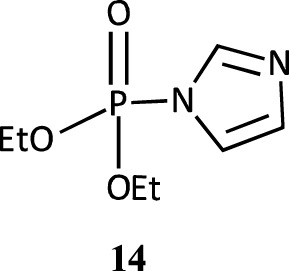


The second-order rate constant for the alkaline hydrolysis of *p*-nitrophenyl diphenyl phosphate in 95% water-5% dioxane (v/v) at 25°C is 3.30 × 10^−1^ M^−1^s^−1^ (Bunton et al., 1968). In 60% water-40% dioxane, under the same conditions, this rate constant was measured as 2.15 × 10^−1^ M^−1^s^−1^, showing decreased rate in the presence of dioxane. This suggests that the TS for the reaction is strongly hydrated, consistent with the low ${∆H}^{‡}$ (9.9 kcal mol^−1^) and significantly negative ${∆S}^{‡}$ (−28 e.u.) values.

Conversely, de Souza and Ionescu (1999) reported the values of the second-order rate constant as 9.6 × 10^−1^ M^−1^s^−1^ and 24.3 M^−1^ s^−1^ in 50% water-50% DMSO and 10% water-90% DMSO (v/v), respectively, clearly showing the accelerating influence of added DMSO. The rate-solvent mixture profile showed very little effect of added DMSO between the 0%–50% DMSO range; there was a steady increase in rate from 50% DMSO to reach 24.3 M^−1^ s^−1^ in 90% DMSO. None of these authors supplied the value of the second-order rate constant in 100% water. From the data of Bunton et al. (1968), a rough extrapolation could be made to give the rate constant in pure water as 2.9 × 10^−1^ M^−1^s^−1^; this value agrees with the value of 2.6 × 10^−1^ M^−1^s^−1^ measured in the laboratories of Williams (Ba-Saif et al., 1990) for reaction in pure water. This implies that the rate of reaction undergoes ca. 4-fold and 100-fold acceleration in 50% water-50% DMSO and 10% water-90% DMSO, respectively; the rate increase in 90% DMSO is therefore modest, when compared to the effects of this solvent in the hydrolysis of phosphate monoester dianions considered earlier. The activation parameters ( ${∆H}^{‡}$ = 9.0 kcal mol^−1^; ${∆S}^{‡}$ = −32.6 e.u.) in 90% DMSO (de Souza and Ionescu, 1999) are comparable to those ( ${∆H}^{‡}$ = 9.9 kcal mol^-1^; ${∆S}^{‡}$ = −28 e.u.) in predominantly aqueous solution (Bunton et al., 1968), suggesting that the same mechanism operates in the two solvent systems.

**FIGURE 7:**
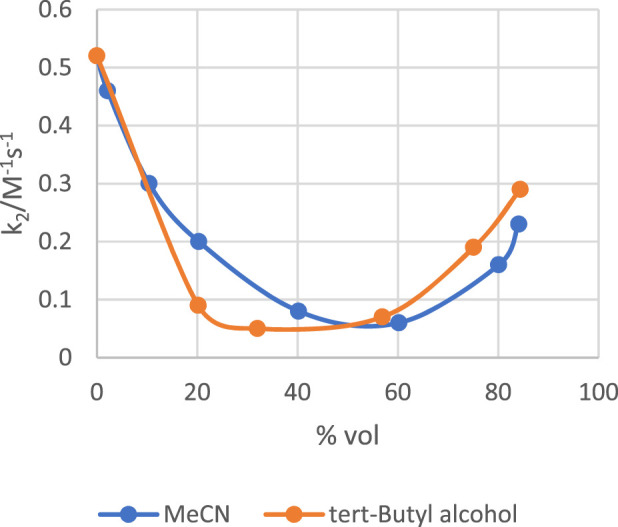
Plots of the second-order rate constant (*k*_*2*_/M^-1^s^-1^) *versus* % volume for the alkaline hydrolysis of *p*-nitrophenyl diphenyl phosphate in water-MeCN and water-*tert*-butyl alcohol mixtures. Data taken from the work of Bunton et al. (1996).

On the other hand, incremental addition of quantities of MeCN or *tert*-butyl alcohol to water retards the rate of the alkaline hydrolysis of *p*-nitrophenyl diphenyl phosphate across the entire range of organic solvent-water mixtures studied to give rate constants that are lower in these water-organic solvent mixtures than in pure water (Bunton et al., 1996), a clearly different scenario from the behaviour in DMSO (de Souza and Ionescu, 1999). Data in water-MeCN and water-*tert*-butyl alcohol mixtures obtained in the study by Bunton et al. (1996), plotted in Figure 7, show that the second-order rate constant, *k*_*2*_, in both solvent mixtures go through a minimum to attain values in ca. 84% organic solvent which are 44% (water-MeCN) and 56% (water-*tert*-butyl alcohol) of the value of *k*_*2*_ in pure water. Bunton et al. have explained that the rate decrease is caused by the stabilization of the phosphate ester substrate through the decrease of its activity coefficient on the addition of the organic solvent to water. This GS stabilization is partially offset by the stabilization of the anionic TS by the organic component of the solvent mixture. The increase in rate in the less aqueous solvent mixtures heralds the destabilization of the GS in this region due to the partial desolvation of the anionic nucleophile. This concept is considered further below (see Section 2.2.4) where solvent effects on the reactivities of phosphate and phosphinothioate esters are discussed.

Although not definitively proven, the accepted mechanism for the hydrolysis of *p*-nitrophenyl diphenyl phosphate in water and predominantly aqueous water-organic solvent mixtures involves the attack of the nucleophile on the substrate to form a pentacoordinate intermediate from which *p*-nitrophenoxide, the leaving group, is expelled (Bunton et al., 1968; Mackay et al., 1987). The reaction of this substrate with the less basic nucleophile, phenoxide, has been shown to be concerted (Ba-Saif et al., 1990). The activation parameters of ${∆H}^{‡}$ = 9.0 kcal mol^-1^ and ${∆S}^{‡}$ = −32.6 e.u. in 90% DMSO (de Souza and Ionescu, 1999) when compared with the values in 5% aqueous dioxane (Bunton et al., 1968), show that the decrease in the activation enthalpy was more important than the decreased activation entropy in going from the predominantly aqueous water-dioxane mixture to 95% DMSO and points to a probable similarity in reaction mechanism in the two solvent systems.

### 2.2 Phosphinates and phosphinothioates

Although there are no known naturally occurring phosphinate and phosphinothioate esters, these synthetic compounds are structurally related to phosphate and phosphonate esters that have found use as pesticides, neurotoxins, and other biologically active and important substances (Toy and Walsh, 1987). A number of suitably substituted phosphinic acids function as metalloprotease inhibitors (Abdou, 2020). Understanding the mechanisms of phospho group transfer reactions involving phosphinates and their thio analogues has often helped to elucidate the constitutional and environmental factors at play in the reactions of phosphonates and phosphates and, by extension, in biological phosphoryl transfer reactions (Hengge and Onyido, 2005).

#### 2.2.1 Reactions with oxygen nucleophiles

Phosphinates (**5a**) and phosphinothioates (**5b**) as esters of phosphinic acid (**15**) represent the simplest structural framework among organophosphorus esters. The proton in the OH group in **15** is replaced by an alkyl group or fragment or an aryl group to form the ester **5**.


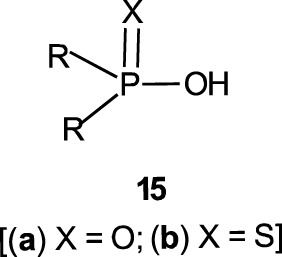



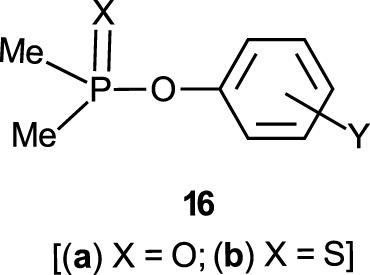



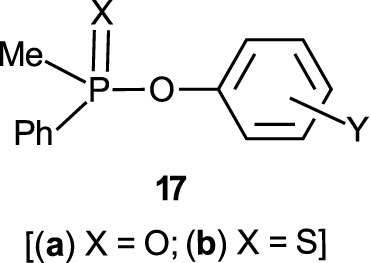



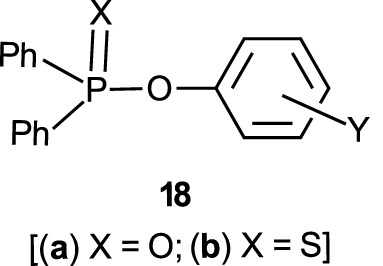


The reactions of phosphinates and phosphinothioates (**16**–**18**) with nucleophiles in a variety of solvents has been the subject of detailed mechanistic studies. Table 1, adapted from our earlier review article (Hengge and Onyido, 2005), summarizes the Brönsted and/or Hammett selectivity parameters reported in the literature for the reactions of oxygen nucleophiles with different phosphinate and phosphinothioate ester substrates with oxygen nucleophiles in different solvents. These selectivity parameters form the basis for the mechanisms assigned to these reactions. Broad mechanistic behaviours are discernible from the body of data summarized in Table 1, depending on the basicity of the attacking nucleophiles and the polarity of the reaction medium.

#### 2.2.2 Resolving mechanistic ambiguities

Two mechanistic ambiguities in the literature which have now been satisfactorily resolved should be pointed out, to aid a discussion of the mechanistic categorizations mentioned above. The first ambiguity concerns entry 13a in Table 1 for the hydrolysis of the aryl dimethylphosphinothioates **16b** in water, first studied by Istomin et al. (Eliseeva et al., 1979) who concluded that the mechanism of the reaction was associative on the basis of a positive value of Hammett ρ ( $\sigma^{0}$ ) = 1.28. A re-evaluation of the data of these workers yielded a Brönsted $\beta_{\mathrm{lg}}$ value which suggests that the reaction occurs via a concerted mechanism in which there is a moderate cleavage of the P-OPhX bond in the TS, prompting a reinvestigation of the system (see entry 13b), to obtain the Brönsted parameters $\beta_{nuc}$ = 0.47 and $\beta_{\mathrm{lg}}$ = −0.53, the Hammett parameter $\left.{\rho (\sigma }^{-}\right)$ = 1.17, and heavy atom kinetic isotope effect (KIE) values of ^*18*^ *k* = 1.0124 and ^*15*^ *k* = 1.0009, leading to the unambiguous conclusion that the reaction is a concerted phospho transfer with moderate bond formation and cleavage in the TS (Onyido et al., 2005b). The reactions of the same substrate with moderately to strongly basic alkoxides as well as with phenoxide ion and other weakly basic phenoxides were shown in the same study (Onyido et al., 2005b) to occur by the concerted mechanism.

The second ambiguity which surrounds entry 15a in Table 1, the hydrolysis of the same substrate **16b** in 50% water-50% ethanol, arose exactly the same way as outlined above for the reaction in water. The reaction was first investigated by the same group, Istomin and Eliseeva (1981) to obtain a Hammett ρ ( $\sigma^{0}$ ) value of 1.72 as evidence for an associative mechanism. A Brönsted linear free energy relationship (LFER) reinvestigation of the same reaction system (Kalu et al., 2022) yielded the Brönsted exponents $\beta_{nuc}$ = 0.35 and $\beta_{\mathrm{lg}}$ = −0.64 and the Hammett parameter $\left.{\rho (\sigma }^{-}\right)$ = 1.54 (see entry 15b), which are consistent with a concerted reaction with substantial dissociative character. As in water, the reactions of this substrate with moderately to strongly basic alkoxides as well as with phenoxide ion and its weaker analogues were all shown to proceed by the concerted mechanism in 50% water-50% ethanol (Kalu et al., 2022).

#### 2.2.3 Reactivity trends

There are three trends that are embedded in or discernible from the data collated in Table 2, which are of mechanistic significance; these are now discussed.

**TABLE 2:** Hammett and/or Brönsted selectivity parameters for the reactions of phosphinate and phosphinothioate esters with oxyanionic nucleophiles in different solvents^a^.

Entry	Substrate	Nucleophile	Solvent	T (^o^C)	Selectivity Parameter(s)	Mechanism	References (see footnotes^d–t^)
1	Ph_2_P (=O)-OPhX	EtO^-^	EtOH	25	$\rho \left(\sigma \right)$ 2.6	Associative	^d^ ^,^ ^e^
2	Ph_2_P (=O)-OPhX	HO^-^	60% H_2_O-40% acetone	25	$\rho \left(\sigma \right)$ 2.20	Associative	^f^
3	Ph_2_P (=O)-OPhX	HO^-^	H_2_O	25	$\rho \left(\sigma^{0}\right)$ 1.40	Associative	^g^
4	Ph_2_P (=O)-OPhX	HO^-^	50% H_2_O-50% EtOH	25	$\rho \left(\sigma^{0}\right)$ 1.93	Associative	^h^
5	Ph_2_P (=O)-OPhX	HO^-^	90% H_2_O-10% dioxane	25	$\rho \left(\sigma \right)$ 1.55	Associative	^i^
6	Ph_2_P (=O)-OPhX	PhO^-^	H_2_O	25	$\beta_{nuc}$ 0.46, $\beta_{\mathrm{lg}}$ -0.79	Concerted	^j^
7	Ph_2_P (=O)-OPhX	PhO^-^	EtOH	25	ρ-σ correlation	Associative	^k^
8	Me_2_P (=O)-OPhX	HO^-^	90% H_2_O-10% dioxane	25	$\rho \left(\sigma^{-}\right)$ 0.93 $\beta_{nuc}$ 0.41, $\beta_{\mathrm{lg}}$ -0.47b	Concerted	^l^
9	Me_2_P (=O)-OPhX	EtO^-^	EtOH	25	$\rho \left(\sigma \right)$ 2.69, $\rho \left(\sigma^{0}\right)$ 2.77	Associative	^m^
10	Ph_2_P (=S)-OPhX	HO^-^	0%H_2_O-50% EtOH	25	$\rho \left(\sigma^{0}\right)$ 1.90	Associative	^n^
11	Ph_2_P (=O)-SPhX	HO^-^	H_2_O-MeOH	25	$\rho \left(\sigma \right)$ 1.46	Associative	^o^
12	PhMeP(=O)-OPhX	EtO^-^	EtOH	25	$\rho \left(\sigma \right)$ 2.71, $\rho \left(\sigma^{0}\right)$ 2.78	Associative	^p^
13a	Me_2_P (=S)-OPhX	HO^-^	H_2_O	25	$\rho \left(\sigma^{0}\right)$ 1.28	Associative	^q^
13b	Me_2_P (=S)-OPhX	HO^-^ & PhO-c	H_2_O	25	$\rho \left(\sigma^{-}\right)$ 1.17 $\beta_{nuc}$ 0.47, $\beta_{\mathrm{lg}}$ -0.53	Concerted	^r^
14	Me_2_P (=S)-OPhX	HO^-^ & PhO-c	70%H_2_O-30% EtOH	25	$\rho \left(\sigma^{-}\right)$ 1.57, 1.52 $\beta_{nuc}$ 0.44, $\beta_{\mathrm{lg}}$ -0.62	Concerted	^s^
15a	Me_2_P (=S)-OPhX	HO^-^	50%H_2_O-50% EtOH	25	$\rho \left(\sigma^{0}\right)$ 1.72	Associative	^q^
15b	Me_2_ (=S)-OPhX	HO^-^ & PhO-c	50%H_2_O-50% EtOH	25	$\rho \left(\sigma^{-}\right)$ 1.54, 1.45 $\beta_{nuc}$ 0.35, $\beta_{\mathrm{lg}}$ -0.64	Concerted	^t^
16	Me_2_P (=S)-OPhX	HO^-^ & PhO-c	30%H_2_O-70% EtOH	25	$\rho \left(\sigma^{-}\right)$ 1.50, 1.29 $\beta_{nuc}$ 0.34, $\beta_{\mathrm{lg}}$ -0.61	Concerted	^t^
17	Ph(Me)P (=S)-OPhX	HO^-^	50%H_2_O-50% EtOH	25	$\rho \left(\sigma^{0}\right)$ 1.83	Associated	^n^
18	Ph(MeSO_2_CH_2_) P (=O)-OPhX-	HO^-^	80%H_2_O-20% dioxane	25	$\rho \left(\sigma \right)$ 1.45	Associated	^u^

^a^ This Table is an expanded version of Table 1 by Hengge and Onyido (2005).

^b^ The value of $\beta_{\mathrm{lg}}=-0.47$ was calculated and reported by Kalu et al. (2022) from the original data published by Douglas and Williams (1976).

^c^ The reactions of moderately to strongly basic alkoxides, as well as those of phenoxide ion and its less basic analogues, with this substrate occur via the concerted mechanism in water and the aqueous ethanolic solvents indicated in this table.

^d^ Dunn and Buncel (1989).

^e^ Pregel et al. (1995).

^f^ Cook and Rahhal-Arabi (1985).

^g^ Eliseeva et al. (1978).

^h^ Istomin et al. (1982).

^i^ Williams and Naylor (1976).

^j^ Bourne et al. (1988).

^k^ Dunn et al. (1990).

^l^ Douglas and Williams (1976).

^m^ Buncel et al. (2004).

^n^ Istomin and Eliseeva (1981).

^o^ Cook and Rahhal-Arabi (1985).

^p^ Onyido et al. (2005b).

^q^ Eliseeva et al. (1979).

^r^ Onyido et al. (2005a).

^s^ Kalu et al. (2021).

^t^ Kalu et al. (2022).

^u^ Cevasco and Thea (1993).

**FIGURE 8:**
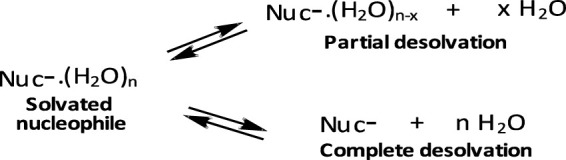
Desolvation of a solvated nucleophile molecule in the GS, giving rise to a more reactive nucleophile; the desolvation may be partial or complete.

First, reactions of the same phosphinate ester substrate with the same nucleophile are slower in ethanol and aqueous ethanol solvents than in pure water, contrary to the prediction of the Hughes-Ingold theory of solvent effects for charge-type 1 S_N_2-type reactions of neutral substrates and negatively charged nucleophiles. For such reactions, destabilization of the nucleophile by desolvation by the less polar organic solvent makes it more reactive, thereby decreasing the reaction activation energy, which results in significant rate increases (Hughes and Ingold, 1935; Ingold, 1969; Parker, 1969; Parker, et al., 1978). Desolvation involves the partial or complete removal, from the nucleophilic site, of solvent molecules associated with the nucleophile in the GS, as illustrated in the equilibria shown in Figure 8. This GS destabilization makes the nucleophile more reactive. The trend for the nucleophilic displacement reactions on phosphorus esters is different because non-polar solvents stabilize the neutral substrates to greater extents than they destabilize the negatively charged nucleophiles (Bunton et al., 1996), leading to rate reductions in such solvents. The means by which less polar solvents stabilize neutral phosphorus esters is discussed further below.

Second, all reactions in water involving phenoxide ion and its weakly basic analogues, all of which are weaker nucleophiles when compared with hydroxide ion, react with the same substrates by a concerted mechanism. The corresponding reactions with the much more basic hydroxide ion as nucleophile occur via the associative mechanism. Table 2 lists the p*K*_*a*_ values showing the relative basicities in water, ethanol and some water-ethanol solvent mixtures of the anionic oxygen nucleophiles employed in the reactions. The same point was made earlier in a study of the reaction of fenitrothion ([*O*,*O*-dimethyl *O*-(3-methyl-4-nitrophenyl) phosphorothioate], **19**) with a variety of oxyanionic nucleophiles–hydroxide, alkoxide, and phenoxides–in water (Omakor et al., 2001) and appears to be general for the reactions of phenoxides and other weakly basic oxyanions with a diversity of (thio)phosphates and (thio)phosphinates for which kinetic data are available. Thus, we have a mechanistic dichotomy in water in which the strongly basic nucleophile hydroxide ion reacts by an associative mechanism, while the weaker nucleophiles react by a concerted mechanism. The question that arises then is, what is responsible for this difference in behaviour of the two oxyanionic nucleophile types which is denominated by nucleophilic strength?


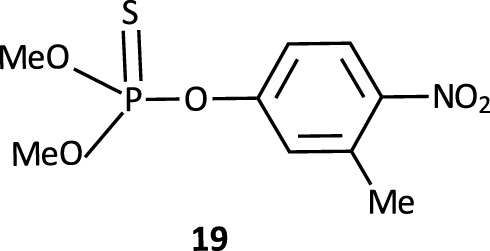


The third trend in Table 2 is that all reactions which proceed by the concerted mechanism in water proceed by the associative mechanism when the solvent is changed to ethanol. This change of mechanism has been explained (Buncel et al., 2004) on the basis of the large changes in the basicity of the nucleophiles, as measured by their ${pK}_{a}$ values, which occur when the solvent is changed from the polar medium water to the less polar solvent ethanol. The p*K*_*a*_s of a number of oxygen and nitrogen bases in water, ethanol and acetonitrile are assembled in Table 3 and it is clear from this table that both types of bases are stronger in ethanol than they are in water.

**TABLE 3:** The p*K_a_* values of selected oxygen and nitrogen bases in water, ethanol, various H_2_O-EtOH solvent mixtures, dimethyl sulphoxide, and acetonitrile.

pKa values in base	H_2_O^a^	EtOH	H_2_O-EtOH 70 : 30^b^	H_2_O-EtOH 50 : 50^b^	H_2_O-EtOH 30 : 70^b^	DMSO	MeCN
Oxygen bases							
Ethoxide	16.0	19.18^c^					
Hydroxide	15.74		16.60	16.75	17.21		
Phenoxide	9.95	15.76^c^	10.54	11.16	11.60		
Nitrogen bases							
n-Butylamine	10.59					11.1^d^	18.26^e^
Benzylamine	9.34					9.81^f^	16.76^e^
Piperidine	11.22					10.6^d^	18.92^e^
Morpholine	8.36					8.94^f^	16.62^e^
Aniline	4.58					3.7^d^	10.56^d^

^a^ Values at 25°C, taken from Jencks and Regenstein (1970).

^b^ Obtained according to the method of Altun and Koseoglu (2013); see also Kalu et al. (2021), Kalu et al. (2022).

^c^ Values at 25°C, taken from Um et al. (1997).

^d^ Taken from Kim et al. (2001).

^e^ Taken from Coetzee (1967).

^f^ Taken from Kolthoff et al. (1968).

The dielectric constant, ε , of water and ethanol are 80.2 and 25.2, respectively, at 20°C (Gregory and Clarke, 2005). In the lower polarity medium, both nucleophiles and leaving groups become stronger bases, making the nucleophiles stronger and the P-Nuc bond easier to form, while the leaving groups become poorer and the P-O bond more difficult to break. These two effects act together to make reactions in the less polar solvent more associative and less dissociative. This change in the mechanistic character of the reaction is possible because there is a positive cross interaction between the nucleophile and the leaving group in the concerted TS, measured (Jencks and Jencks, 1977; Jencks, 1985) by the cross-interaction coefficient, $p_{xy}$ , defined in Eq. 1.

$$p_{xy}=-\frac{\partial \beta_{\mathrm{lg}}}{\partial pK_{nuc}}=\frac{\partial \beta_{nuc}}{\partial pK_{\mathrm{lg}}}=\frac{\partial^{2}\log k}{\partial pK_{\mathrm{lg}}\partial pK_{nuc}}$$ (1)

The above reasoning to explain the change in the mechanistic character observed in the reactions collected in Table 2 with solvent change from water to ethanol is tantamount to the assumption that, for any particular concerted reaction, e.g., the hydrolysis of the phosphinothioate esters Me_2_P (=S)-OPhX (entry 13b) for which $\beta_{nuc}$ 0.47 and $\beta_{\mathrm{lg}}$ -0.53 are the Bronsted parameters, there exists a spectrum of transition states, spanning from the TS at X in Figure 1 towards the associated end of the diagram, as shown by the trajectory (D) defined by the arrows in red. This continuum of transition states is obtained by the sequential tightening of the P-O bond which results from the corresponding increases in the base strengths of the nucleophile and leaving group; this tightening moves the concerted TS in incremental steps towards the associated pentacoordinate structure. However, a drawback in this rationalization is that it omits the significant difference in polarity, ∆ε = 55, between water and ethanol.

**FIGURE 9:**
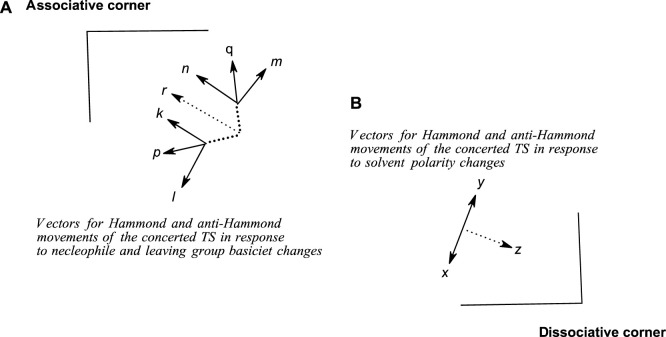
A schematic diagram showing Hammond and anti-Hammond vectorial movements of the concerted TS in response to changes in **(A)** nucleophile and leaving group base strengths and **(B)** solvent polarity.

This path to associative structures is quantified by a vector *r*, which is the resultant of the TS movement due to changes in nucleophile basicity (quantified by vector *p*) and that of the TS movement due to changes in leaving group basicity (quantified by vector *q)*. Vector *p* is the resultant vector of the Hammond and anti-Hammond movements of the TS resulting from changes in nucleophile basicity (vectors *k* and *l*, respectively, Figure 9), while vector *q is* the resultant vector of the Hammond and anti-Hammond movements of the TS resulting from changes in leaving group basicity (vectors *m* and *n*, respectively, Figure 9). Figure 9 gives a diagrammatic representation of how these vectors interact to effect TS movements as a consequence of nucleophile and leaving group basicity changes, in tandem with solvent polarity changes (see below).

An examination of the combined influence of the change in the base strengths of the nucleophile and leaving group and change of solvent polarity as the solvent is changed from water to ethanol on the structure of the concerted TS was undertaken by studying the same reaction in a series of water-ethanol mixtures in which the solvent polarity was decreased by sequentially increasing the proportion of the ethanol component (Onyido, et al., 2005a; Kalu et al., 2021; Kalu et al., 2022). The outcome of this study was counterintuitive: the TS, rather than move towards the associative end of Figure 2, moved in the opposite direction, towards the dissociative end, up to a point Y where further decreases of solvent polarity had no effect on TS structure. This movement towards the dissociative end of Figure 2 is shown by the trajectory (E) defined by the arrows in blue and quantified by a vector *z*, which is the anti-Hammond response to the movement of the concerted TS along the reaction coordinate according to the arrow *x* ↔ *y* (Harris et al., 1979; Jencks, 1981; Herschlag and Jencks, 1987) in response to solvent polarity changes. This movement occurs because increasing the quantity of the less polar solvent (ethanol) stabilizes the phosphorus ester substrate more than it destabilizes the negatively charged nucleophile (Bunton et al., 1996) and more than it stabilizes the TS, thereby effecting a nett decrease in the energy of the GS. The TS moves towards the stabilized end of the diagram as its consequential Hammond response to the nett stabilization of the GS. The anti-Hammond effect is the movement of the TS in a direction perpendicular to the reaction coordinate, shown by the vector z, towards more dissociative structures.

As can be seen from the above analysis, increasing the base strength of the nucleophile and leaving group moves the concerted TS, along vector *r*, towards associative TS structures, while a decrease in solvent polarity effectuates TS movement along vector *z*, towards the dissociative end of the diagram. For the situation in which both nucleophile and leaving group basicity is increased and solvent polarity is decreased at the same time by the incremental enhancement of the proportion of ethanol in the aqueous ethanol solvent, the observed movement of the TS toward more dissociative structures is the balance between two effects. The various scenarios inherent in the relative magnitudes of the two opposing vectors *r* and *z* are summarized in Table 4.

**TABLE 4:** The relative magnitudes of the vectors r and z in Figure 2 and their effects on the structure of the concerted TS for the reaction of 8b with HO^-^ and PhO^-^ nucleophiles in aqueous ethanol solvents (entries 12b, 13, 14b, and 15 in Table 1). From Kalu et al. (2022), reproduced by permission of the Royal Society of Chemistry.

Relative magnitudes of vectors r and z	r > z	r < z	r = z
Resultant of opposing vectors	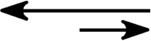	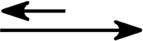	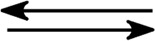
Effect on TS structure	TS becomes more associative (i.e., tighter) in character	TS becomes more dissociative (i.e., looser) in character	TS structure is unchanged
Solvent	Pure ethanol^a^	30% ethanol^b^	50% ethanol or higher % ethanol mixtures^c^

^a^ See Buncel et al. (2004).

^b^ See Kalu et al. (2021).

^c^ See Kalu et al. (2022).

#### 2.2.4 The origin of observed solvent polarity effects on reaction rates

**FIGURE 10:**
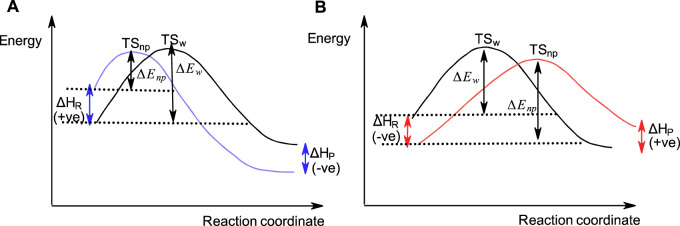
A hypothetical reaction coordinate diagram showing qualitatively the S_N_2-type reactions in water and a less polar organic solvent involving an anionic nucleophile with **(A)** a carbon-based substrate, and **(B)** a phosphinate ester substrate. In **(A)**, the initial state is destabilized in the less polar solvent ( $∆H_{R}$ is positive) to result in an earlier TS and faster reaction ( $∆E_{np}$ < $∆E_{w}$ ) in this solvent than in water. In **(B)**, the initial state is stabilized in the less polar solvent ( $∆H_{R}$ is negative) to result in a later TS and a slower reaction ( $∆E_{np}$ > $∆E_{w}$ ) when compared with the rate in water.

As pointed out above, S_N_2-like reactions of phosphinates (Cook et al., 1986b; Blásko et al., 1991; Bunton et al., 1996) and some other organophosphorus esters (Blásko et al., 1991) with negatively charged nucleophiles in water become slower when conducted in less polar solvents. This is contrary to the general solvent effects encountered in the reactions of carbon-based S_N_2 reactions which proceed faster in less polar solvents than in water, in accord with the Hughes-Ingold theory of solvent action. This behaviour, which was investigated in some depth (Blásko et al., 1991; Bunton et al., 1996), has been attributed to the stabilization of the neutral ester substrate by the less polar solvent, an inhibitory effect that more than compensates the rate-increasing destabilization of anionic nucleophiles due to desolvation by the organic solvent. Figure 10 shows the reaction coordinate diagrams for (A) a hypothetical S_N_2 reaction which obeys the Hughes-Ingold theory of solvent action, in which the organic solvent destabilizes the initial state by desolvating the nucleophile, and for (B) a hypothetical S_N_2(P) reaction in which the organic solvent stabilizes the initial state by decreasing the activity coefficient of the neutral organic substrate (see below).

Bunton et al. (1996), in their study of the rates of hydrolysis of diverse organophosphorus ester substrates in two binary aqueous organic solvents (water-MeCN and water-*tert*-butyl alcohol), demonstrated linearity with a negative slope in plots of substrate activity coefficient, $\gamma_{s}$ , *versus* mole fraction of solvent, $\chi_{solv}$ , according to Eq. 2, where $K_{s}$ is the slope, an empirically determined quantity.

$$\log\gamma_{s}=-K_{s}\chi_{solv}$$ (2)

This qualitative treatment, which can be extended to other binary aqueous organic solvents in which the organic solvent is less polar than, and miscible, with water, is sufficient to rationalize why organophosphorus esters behave differently from alkyl substrates in their S_N_2 reactions with anionic nucleophiles.

#### 2.2.5 Reactions with nitrogen nucleophiles

**FIGURE 11:**
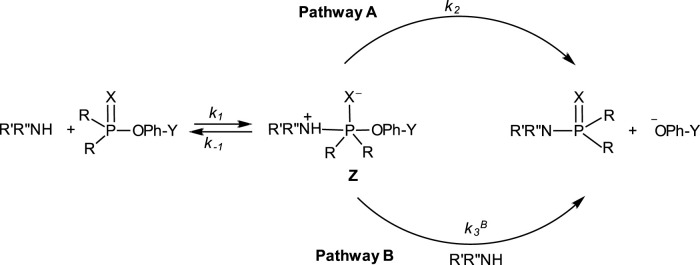
The mechanisms for the aminolysis of aryl phosphinates and aryl phosphinothioates by a diversity of amine types–primary, secondary, and di-amines–in acetonitrile. See Cook et al. (1986a).


In their study of the reactions of a number of aryl phosphinates and aryl phosphinothioates with an array of primary, secondary and diamine nucleophiles in acetonitrile, Cook et al. (1986a) demonstrated the nucleophilic reactivity order of diamine > primary amine > secondary amine. While the diamines and secondary amines obeyed a rate law which is first-order in amine, the kinetics of the only primary amine studied, n-butylamine, obeyed a two-term rate law according to Eq. 3, the first and second terms being first-order and second-order, respectively, in amine. This kinetic behaviour is consistent with the mechanism shown in Figure 11, a conclusion that is reinforced by the observed leaving group effects, solvent effects and activation parameters. In this mechanism, Pathway A is followed by the reactions involving the diamines and secondary amines in which the zwitterionic pentacoordinate intermediate **Z** decomposes unimolecularly to give the reaction products. Although both amine types obey the same rate law, the intermediates arising from them differ in their decomposition modes to obtain the products. For the secondary amines, rate-limiting dissociation of **Z** followed by proton loss, or rate-limiting intramolecular proton transfer to X^−^ which is in concert with leaving group expulsion, are relatable to the measured activation entropy in the region of −59 e.u. (for piperidine, as an example) and a good correlation of $k_{obs}$ with amine ${pK}_{a}$ . On the other hand, with diamines as nucleophiles, **Z** decomposes via intramolecular general base catalysis. Pathway B, which is a general base-catalysis mechanism, is followed by the reaction of *n*-butylamine. The behaviour of n-butylamine is consistent with the rate law observed for its reaction, which is second-order in amine. The TS depicted as **20** has been proposed on the basis of a value of $∆S^{‡}$ = −80 eu at 25°C, for the rate-limiting separation of the leaving group from **Z,** with concurrent proton loss to the second amine molecule. Alternatively, **Z** could be visualized as undergoing concurrent intramolecular transfer to X^−^ with phenoxide expulsion, subsequent to proton transfer to the second amine molecule. Neither of these alternatives could be ruled out on the basis of the data available.

$$k_{obs}=k_{2}\left[amine\right]+k_{3}^{B}{\left[amine\right]}^{2}$$ (3)

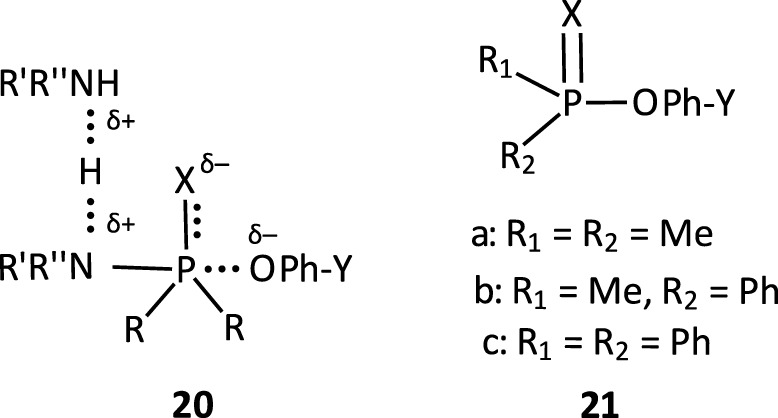


The reactions of anilines with aryl dimethyl, methyl phenyl, and diphenyl phosphinates (**21a**-**c**) in DMSO occur with normal primary deuterium kinetic isotope effects ( $\frac{k_{H}}{k_{D}}$ = 1.05–1.17 for **21a**, 1.15–1.29 for **21b**, and 1.24–1.51 for **21c**) and cross-interaction coefficients (CICs), $\rho_{xy}$ = 0.37, 0.34, and 0.65 for **21a**, **21b**, and **21c**, respectively (Dey et al., 2011). These parameters were interpreted as being consistent with a stepwise mechanism in which the expulsion of the leaving group from the pentacoordinate intermediate was rate-limiting.

Contrary to the above results in acetonitrile and DMSO, Um et al. (2006); Um et al. (2007); Um et al. (2009) have reported that the reactions of 2,4-dinitrophenyl diphenylphosphinate and some other aryl diphenylphosphinate substrates and their thio analogues with secondary amines in the predominantly aqueous binary solvent 80 mol% water-20 mol% DMSO proceed through a concerted mechanism, on the basis of the Brönsted and Yukawa-Tsuno parameters, $\beta_{nuc}$ = 0.32–0.52 and r ∼ 0.3, respectively, measured for the reactions. The reactions of aryl diphenyl phosphinothioates are slower and proceed through tighter transition states than their phosphinate counterparts (Um et al., 2007).

The dielectric constants, ε , of water, MeCN and DMSO are 80.2 (Gregory and Clarke, 2005), 35.9 (Kelly, et al., 2007), and 46.5 (Kelly, et al., 2007), respectively. A value of ε = 76 has been estimated for 80 mol% water-20 mol% DMSO from literature data (Zhuang et al., 2021) while ε for 79 mol% water-21% DMSO was measured as 74.9 (Luzar and Stefan, 1990; Das and Tembe, 1999). These dielectric constant values show that water and the predominantly aqueous 80 mol% water-20 mol% DMSO mixture are substantially more polar than the dipolar aprotic solvents DMSO and MeCN. The p*K*_*a*_ values of the amines used in the studies discussed above in water, MeCN, and DMSO are listed in Table 2. While the p*K*_*a*_s of the amines are consistently higher in MeCN than in water, they are roughly of the same magnitude in water and DMSO. It is apparent from the foregoing that the mechanisms of the reactions involving the amines in Table 2 are more dependent on solvent polarity than on nucleophile basicity.

This phenomenon, in which the mechanism of the reactions of nitrogen nucleophiles with phosphinates and phosphinothioates changes from concerted in water and predominantly aqueous DMSO solvent to associative in the less polar solvents MeCN and DMSO, was also noted and extensively discussed for the reactions of oxygen nucleophiles above, where the change of solvent is from water to the less polar ethanol. Overall, the potential energy surface (PES) involving concerted TS structures should be markedly different from the PES involving associative TS structures. Noting that even though ethanol, MeCN and DMSO all favour the associative mechanism for the reactions of these substrates with oxygen and nitrogen nucleophiles, MeCN and DMSO are dipolar aprotic solvents while ethanol is a hydroxylic one, it appears that the choice of the PES in these reactions is more solvent polarity-driven and is independent of solvent type.

## 3 Discussion

In order to assess the status of information on solvent and solvation effects on reactivities and mechanisms in the phospho group transfers considered in this paper, with the objective of determining future directions for research in this domain, we have summarized available information and data discussed in the preceding sections in Table 5.

**TABLE 5:** A summary of the effects of solvent change on reactivities and mechanism in some of the different reaction systems encountered in phospho group transfers.

Substrate type	Reaction system	Effect of solvent change on reactivity	Effect of solvent change on mechanism	References (see footnotes)
Phosphate monoesters	*p*-Nitrophenyl phosphate monoanion solvolysis in water	Reaction is 14-fold and 16-fold slower in tert-butyl alcohol and tert-amyl alcohol, respectively, due to increased ${∆H}^{‡}$	No effect on mechanism	^a^ ^,^ ^b^
	*p*-Nitrophenyl phosphate monoanion alkaline hydrolysis	Reaction is slower in 80% DMSO-20% aqueous formate buffer, pH = 3.2 due to disfavoured pre-equilibrium proton transfer	Mechanism is same as in water	^c^
	*p*-Nitrophenyl phosphate dianion solvolysis in water	Reaction is 7.5 × 103-fold and 8.8 × 103-fold faster in tert-butyl alcohol and tert-amyl alcohol, respectively, exclusively due to entropic factors	Higher ${∆S}^{‡}$ values of 24.3 e.u. (tert-butyl alcohol) and 23.0 e.u. (tert-butyl alcohol), *cf.* 3.5 e.u. in water, support a change of mechanism, from ANDN SN2(P)-type to DN + AN SN1(P)-type	^a^ ^,^ ^b^
	*p*-Nitrophenyl phosphate dianion alkaline hydrolysis	Reaction increases 106–107 fold in 95% aqueous DMSO or HMPA due to significant GS desolvation and TS stabilization by charge dispersal. Results show that hydrolysis rates substrates with pKa < pKa of are slower in aq. DMSO than water	No change of mechanism was detected from heavy atom KIE studies	^c^ ^,^ ^d^ ^,^ ^e^
	*p*-Nitrophenyl phosphate dianion alkaline hydrolysis	Reaction is accelerated >106 times in MeCN containing 0.02 M water	No change in mechanism occurs	^f^
	Neopentyl phosphate hydrolysis	Reaction is remarkably accelerated 2.5 × 1012-fold in cyclohexane; this is entirely entropic in origin, ascribed destabilization of the ground state and greater charge delocalization in the TS.	No suggestion of change in mechanism	^g^
Phosphorothi oate mono esters	*p*-Nitrophenyl phosphorotioate monoanion alkaline hydrolysis	Reaction is slightly decreased by increasing DMSO quantities. In 95% aq. DMSO, the less favourable ${∆S}^{‡}$ , compared to water, is not counterbalanced by the reduced ${∆H}^{‡}$	No change in mechanism	^h^
	*p*-Nitrophenyl phosphorotioate dianion alkaline hydrolysis	Reaction is accelerated 106 fold in 95% DMSO relative to its rate in water; this dramatic rate increase is enthalpic in origin, ${∆∆H}^{‡}$ = 14.1 kcal/mol	Same mechanism in water	^h^
Phosphate diesters	Bis(*p*-nitrophenyl) phosphate alkaline hydrolysis	Rate first decreased in the 0–70 vol% DMSO region, then increased sharply to reach 15-fold its value in water in 94% DMSO. Dioxane and MeCN gave similar results	No information on mechanism in the aq. organic solvents	^i^
	Dineopentyl phosphate hydrolysis	Rates accelerated 2 × 109-and 5 × 105-fold in cyclohexane and acetone, respectively, relative to water. These accelerations are mainly enthalpic in origin, with minor entropic contributions	Mechanism is same (associative) in water and these solvents	^j^
Phosphate triesters	Diethyl 2,4-dinitrophenyl phosphate hydrolysis	Under nucleophilic catalysis by imidazole and 1-methyl-imidazole, hydrolysis rate imidazole, hydrolysis rate is increased 103-fold and 2 × 102-fold, respectively, in 100% DMSO. Rate increase is caused by decreased solute-solvent interactions in DMSO-rich and pure DMSO solvents	Mechanism for the formation of the phosphorylimidazole intermediate in water is concerted	^k^ ^,^ ^l^
		Alkaline hydrolysis of diethyl 2,4-dinitrophenyl phosphate In water is accelerated ca. 100-fold in 90% DMSO. Decrease in ${∆H}^{‡}$ is more important than decrease in ${∆S}^{‡}$ . Addition of MeCN or tert-butyl alcohol decreased rates to values lower than in water. These solvents stabilize GS better than TS.	Mechanism is associative in both water and 90% DMSO.	^m^ ^,^ ^n^ ^,^ ^o^ ^,^
				^p^
Phosphinates and phosphino-thioates	Hydrolysis of diphenyl aryl phosphinates	Rates of hydrolysis are slower in aq. ethanol, aq. dioxane, and aq. acetone than in water. With p-nitro-phenyl as leaving group, rate in water is 3- to 10-fold faster than in the mixed solvents. GS is slightly better stabilized than TS in mixed solvents	Mechanism is same (associative) in water and these aq. organic solvents	^q^ ^,^ ^r^ ^,^ ^s^ ^,^
	Reactions of amines with diphenyl aryl phosphinate and aryl phosphinothioate substrates	Structural diversity among substrates and amines preclude reactivity comparisons. However, the reaction of piperidine with p-nitrophenyl phosphinate is ca. 100-fold faster in 80 mol% H_2_O-20 mol% DMSO than in MeCN.	Reactions with a variety of amines is associative in MeCN but concerted in 80 mol% H_2_O-20 mol% DMSO.	^t^ ^,^ ^u^ ^,^ ^v^
				^w^ ^,^ ^x^
	Hydrolysis of dimethyl aryl phosphinothioates	Rates of hydrolysis are slower in aq. ethanol solvents than in pure water. With p-nitrophenyl as leaving group, rate in water is 360-fold faster than in 70% aq. ethanol. Higher proportion of ethanol leads to greater stabilization of the GS.	Mechanism is concerted in water and in the aq. ethanol solvents but the TS is looser in the latter set of solvents	^y^ ^,^ ^z^ ^, a’^
	Reactions of dimethyl aryl phosphinothioates with some alkoxides and phenoxides	Rates of reactions of these nucleophiles are slower in aq. ethanol solvents than in pure water. With p-nitrophenyl as leaving group, rates in water are 102–103-fold faster than in 70% aq. ethanol	Mechanism is concerted in water and in the aq. ethanol solvents but the TS is looser in the latter set of solvents	^y^ ^,^ ^z^ ^, a’^

^a^ Kirby and Varvoglis (1967).

^b^ Hoff and Hengge (1998).

^c^ Abell and Kirby (1986).

^d^ Grzyska et al. (2002).

^e^ Sorensen-Stowell and Hengge (2006).

^f^ Beltran et al. (1985).

^g^ Stockbridge and Wolfenden (2009).

^h^ Catrina and Hengge (1999).

^i^ Gomez-Tagle et al. (2006).

^j^ Stockbridge and Wolfenden (2010).

^k^ Orth et al. (2011).

^l^ Campos et al. (2015).

^m^ Bunton et al. (1968).

^n^ de Souza and Ionescu (1999).

^o^ Mackay et al. (1987).

^p^ Bunton et al. (1996).

^q^ Istomin et al. (1982).

^r^ Williams and Naylor (1976).

^s^ Cook and Rahhal-Arabi (1985).

^t^ Cook et al. (1986a).

^u^ Dey et al. (2011).

^v^ Um et al. (2006).

^w^ Um et al. (2007).

^x^ Um et al. (2009).

^y^ Onyido et al. (2005b).

^z^ Kalu et al. (2021).

^a’^ Kalu et al. (2022).

A number of general points emerge from a careful scrutiny of Table 5.

1. For the same nucleophile, the effect on reactivity of solvent change from water to dipolar aprotic solvent (DMSO or HMPA or MeCN or acetone)-water mixtures or to neat dipolar aprotic solvents follows the following order of substrates: phosphate monoester dianions >> phosphate monoester monoanion ≈ phosphate diester monoanion. This points clearly to the role of charges on the substrates in contributing to substantial solvent effects on reactivity. From this perspective, it will be instructive to undertake systematic studies which seek to unravel any inherent quantitative or semi-quantitative relationships between the amount of charge in the GS of a phospho transfer reaction and the magnitude of the rate enhancement observed when the reaction is transferred from water to an aprotic solvent.
2. For the same substrate, the effect of a change from a polar solvent to less polar one is by far greater for charged nucleophiles such as HO^−^ (e.g., the 10^6^–10^7^-fold increase in the rate of the alkaline hydrolysis of phosphate monoester dianions in aq. DMSO or HMPA) than for uncharged nucleophiles such as water (e.g., the ∼ 10^4^-fold or less increase in the solvolysis rate of phosphate monoester dianions in *tert*-butyl alcohol and *tert*-amyl alcohol).
3. The greatest effects are observed when the solvent is changed from water to the much less polar cyclohexane, as found in the hydrolysis of neopentyl phosphate dianion (10^12^-fold rate increase) and dineopentyl phosphate monoanion (10^9^-fold rate increase) in cyclohexane saturated with water. An estimation of the magnitude of the rate enhancement experienced by phospho transfer reactions involving specific substrate types (phosphate monoanion or dianion) as functions of solvent polarity as measured by, e.g., solvent dielectric constant, will therefore be an important contribution to the understanding of solvent effects in these processes.
4. With phosphate or phosphorothioate monoester monoanions as substrates for the alkaline hydrolysis reactions, the effects of solvent change, from water to dipolar aprotic solvents or their aqueous mixtures, on reactivity are quite modest, usually in the range of 10- to 20-fold increase in rates.
5. Rate increases which are also modest, in the range of 10^2^-10^3^-fold, are induced by solvent change, from water to less polar solvents, when the substrates are neutral esters, as encountered in the hydrolysis of phosphate triesters and in the reactions of phosphinate/phosphinothioate esters with amines.
6. All reactions of the nucleophiles hydroxide, alkoxides and phenoxides with phosphinate and phosphinothioate esters are slower in water-ethanol mixtures than in water.
7. It is not in all cases that the activation parameters for the reactions in the solvents of interest were determined in the studies summarized in Table 5. The determination of activation parameters and the thermodynamic parameters for the transfer of solutes from water to the less polar solvents are minimum but vital pieces of information which solvent effect studies should seek to convey.
8. It is instructive to note that for systems for which definitive information about their mechanisms of reaction is available, only in a few cases have the observed solvent effects on reactivities delivered changes in mechanism as well.
9. This review has revealed that scant attention has been paid to solvent effects on catalyzed reactions. It will be an important endeavour to investigate the relationship between the effect of catalysts and solvents on reactivity and mechanisms when compared with uncatalyzed systems.

From the foregoing, it is clear that solvent effects, which are deserving of attention because the rate enhancements they deliver approach or surpass those found in enzymatic reactions, have been encountered only in a few of the phospho group transfers considered in this review. Such interesting systems involve a few phosphate monoester dianions and phosphate diester monoanions as substrates while the solvents utilized are aprotic solvents such as cyclohexane, DMSO and a few other dipolar aprotic solvents which are, to different extents, less polar than water. The extension of such studies to more phosphate ester substrates of different activation statuses is critical to characterising a good range of responses to solvent changes, which is a prerequisite to the proper understanding of the causative factors that deliver large rate enhancements. The combination of negative charges in the GS with the hydrophobic environment which solvents with reduced dielectric constants relative to water provide, in order to obtain significant rate enhancements, suggests the importance of GS desolvation and the stabilization of the more charge delocalized TS in the enactment of “enzyme”-type rates in such systems. Such charge redistribution, which is intensely promoted in the non-polar active sites of enzymes through preferential charge destabilization in the GS and stabilization of the redistributed charges in the TS, is behind the proposal that desolvation contributes to rate enhancements by enzymes.

## 4 Concluding remarks

The authoritative computational work by Warshel’s group (Warshel et al., 2006) does not support desolvation as a mechanism by which enzymes enact tremendous rate enhancements. Their work demonstrated that enzyme active sites supply preorganized environments with electrostatic endowments for TS stabilization that far outweigh the TS stabilization which obtains in water. Polar interactions are promoted in the hydrophobic environment of the enzyme, compared to water, by the enhanced strength of these interactions which is known to increase with the reciprocal of the effective dielectric constant of the active site environment (Richard et al., 2014). Nevertheless, it is important for solution experimentalists to undertake detailed evaluations of solvent effects in the reactions of activated and less activated substrates so as to systematize and quantify GS desolvation and TS stabilization effects by solvents in order to fully understand what happens when the reactants and transition states are transferred from water to less polar environments. Such studies will be important contributions to the physical organic chemistry of phospho group transfer. Such studies will also seek to provide answers to the important question posed by Stockbridge and Wolfenden (2009) about what changes occur in the TS of a phospho transfer reaction and the degree to which water molecules continue to be associated with the phosphate ester substrate when it is transferred to more hydrophobic environments.
